# Computational design of phosphate fluoride cathode materials for Na-based batteries

**DOI:** 10.1039/d5ta04213e

**Published:** 2025-09-15

**Authors:** Hafssa Arraghraghi, Michael Häfner, Matteo Bianchini

**Affiliations:** a Department of Biology, Chemistry, and Geosciences, University of Bayreuth Universitätsstrasse 30 95445 Bayreuth Germany matteo.bianchini@uni-bayreuth.de; b Bavarian Center for Battery Technology (BayBatt) Weiherstrasse 26 95447 Bayreuth Germany

## Abstract

Na-ion batteries are sustainable, low-cost alternatives to Li-ion batteries. However, their limited energy density has hindered a widespread adoption. Among positive electrode materials, polyanionic compounds approaching the performances of LiFePO_4_ are being investigated. The Na_3_V_2_(PO_4_)_2_F_3_ family of phosphate fluorides in particular has demonstrated sufficient specific capacity at high operating voltage. Combined with remarkable capacity retention and power capabilities, it entered applications in power tools. However significant concerns exist about the availability of vanadium. To find alternatives, we explored the substitution of V with other transition metals. We considered Ti, Cr, Mn, Fe, Co, Ni, Mo, Zr and Nb using first-principles calculations based on density functional theory with the r^2^SCAN functional. For all compounds, we investigated in detail the expected operational voltage, as well as the structural characteristics and Na^+^ mobility *via* nudged-elastic band calculations (NEB). Most metals yield too high voltages for operation within the stability window of common electrolytes, with the notable exceptions of Mn and Mo that show promising voltages over the reversible (de)intercalation of 3 Na/f.u. In all cases, the electrochemical operation is found to occur with small volume change (maximum 6% for Mn) and the computed migration barriers remain similar to vanadium's ones. Finally, we propose potential synthesis reactions for all compounds and calculate their Gibbs free energy. The never-before reported Co-, Mn- and Mo-based compounds are predicted to be synthesizable. Our work suggests the existence of novel promising positive electrode materials for Na-ion batteries, and it suggests potential synthetic routes to experimentally achieve them.

## Abstract 2

Transition metal substitution in Na_*x*_V_2_(PO_4_)_2_F_3_ (0 ≤ x ≤ 4) is investigated using DFT (r^2^SCAN + U), revealing voltage profiles, crystal structures and Na^+^ migration barriers. Potential synthesis routes to the novel materials are evaluated.

## Graphical abstract

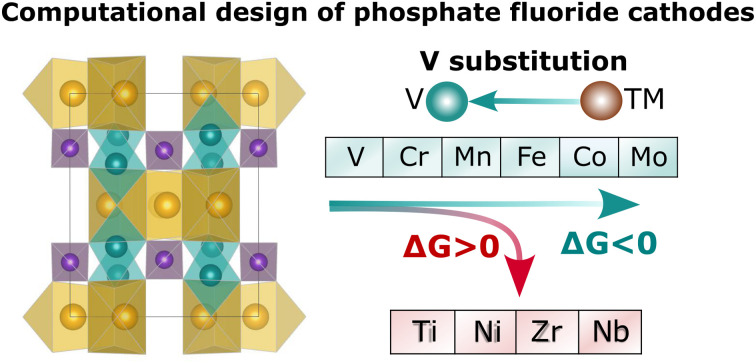

## 1 Introduction

The quest for sustainable energy storage systems is driven by the strong need to exploit renewable energy sources and enable electric vehicles (EVs).^1–3^ Sodium-ion batteries (SIB) have emerged as a promising alternative to lithium-ion batteries (LIB) due to the abundance of sodium and to its low cost compared to lithium.^4–6^

So far, SIB's energy density has been limited especially by the lack of high capacity cathode materials. Among the existing ones, the polyanionic phosphate fluoride family Na_*x*_V_2_(PO_4_)_2_F_3−2*y*_O_2*y*_ with (0 ≤ *y* ≤ 1)^7–14^ is one of the most examined ones. These materials have demonstrated superior performance to layered oxides and are being developed as competitive sodium-ion cathode materials.^15–18^ Na_*x*_V_2_(PO_4_)_2_F_3_ (NVPF) has been the first reported member of the Na_*x*_V_2_(PO_4_)_2_F_3−2*y*_O_2*y*_ family. It stands out due to its electrochemical properties with relatively large specific reversible capacity (128 mA h g^−1^),^17,19,20^ and long cycle life of several thousands of cycles.^21,22^ NVPF also operates at a high average potential^11,19,20,23^ of approximately 3.95 V *vs.* Na^+^/Na, which enables a gravimetric energy density of 507 Wh kg^−1^.^13,17,23,24^ These promising features, in conjunction with the outstanding rate capability, made NVPF the material of choice for the first commercialization by the TIAMAT startup. However, NaxV_2_(PO_4_)_2_F_3_ still faces some issues: firstly, its energy density is, at least theoretically, lower than the one of most layered oxides. Secondly, one last sodium could be theoretically accessed in the structure, but the potential that is required to extract it from Na_1_V_2_(PO_4_)_2_F_3_, which corresponds to the activation of the V^4+^/V^5+^ redox couple, lies at a voltage of 4.9 V *vs.* Na^+^/Na, typically inaccessible within the stability window of common electrolytes.^25,26^ Moreover, strong Na^+^/vacancy ordering impedes ion transport at stoichiometric compositions such as Na_1_V_2_(PO_4_)_2_F_3_.^25^ However the most serious issue related to NVPF is that V is not abundant: it has roughly the same abundance as Ni in the Earth's crust, but a much less developed supply chain. Therefore, it would be desirable to replace V in the Na_3_V_2_(PO_4_)_2_F_3−2*y*_O_2*y*_ (0 ≤ *y* ≤ 1) structural framework with alternative redox active elements. For example, we recently demonstrated the possibility of preparing mixed Fe–V materials and evaluated their performance.^27^

Our work aims to assess elemental substitutions for V in the NVPF-type framework, and potentially lead to positive electrode materials with appropriate voltage windows and Na mobility. Van der Lubbe *et al.*^28^ recently showed that Na_3_V_2_(PO_4_)_2_F_3_ can be substituted by W, Mo and Nb, potentially leading to lower operating voltages. Here we expand the selection of elements across 3d and 4d transition metals, and also propose synthesis routes to access the novel materials. Attention is devoted to observe whether the desodiation occurs within the stability window of common electrolytes, and in particular whether the third plateau (extraction of Na from Na_1_VPF to VPF) can be lowered, or if the Na_4_–Na_3_ one can be increased, either of which would represent a significant increase in specific capacity and energy.

As first step, we re-examine Na_*x*_V_2_(PO_4_)_2_F_3_ (0 ≤ *x* ≤ 4) using first-principles calculations with the recently-developed r^2^SCAN metaGGA functional, using D4 dispersion and U Hubbard corrections.^29^ The r^2^SCAN functional provides improved accuracy in describing structural properties, energetics, and electronic correlations.^30^ Its non-empirical formulation makes it well suited for transition metal-based compounds and polyanionic frameworks. For the substituted compounds, we investigate diffusion mechanisms using the nudged elastic band (NEB) method. Given our recent work, where we reported new experimental and computational finding regarding the solid-state synthesis of Na_3_V_2_(PO_4_)_2_F_3−2*y*_O_2*y*_ compounds,^19^ we finally also analyze possible synthesis routes to the novel computed materials. The Gibbs free-energy computed as a function of temperature is evaluated to assesses the feasibility of the proposed synthesis approaches.

## 2 Methods

The Na_*x*_M_2_(PO_4_)_2_F_3_ (0 ≤ *x* ≤ 4)-type structure under investigation was simulated with varying concentrations of sodium. M represents a selection of chemical elements from the first and second row of transition metals, respectively Ti, V, Cr, Mn, Ni, Fe, Co, Mo, Nb, Zr, and *x* entails five different concentrations of sodium (0, 1, 2, 3, 4). All calculations were conducted using the Vienna *Ab initio* Simulation Package VASP,^31,32^ employing augmented plane-wave pseudo-potentials,^33^ the r^2^SCAN exchange-correlation functional, and the approximate D4 dispersion correction.^29^ Hubbard correction was used for the following transition metals and their respective U values, as evaluated by Swathilakshmi *et al.*:^34^ Ti (2.3 eV), V (1 eV), Cr (1.8 eV), Mn (2.1 eV), Ni (1.8 eV), Fe (3.1 eV), Co (1.8 eV). For Mo (0.57 eV), and Nb (0.71 eV) we determined the Hubbard correction values through a benchmarking study by comparing the calculated formation energies for NbO_2_, Nb_2_O_5_, MoO_2_, and MoO_3_ with experimental JANAF values^35^ as presented in Table S1, Fig. S1 and S2 in the SI.

The initial geometry was derived from experimental crystallographic data for the Na_*x*_V_2_(PO_4_)_2_F_3_ (0 ≤ *x* ≤ 4). The structure for Na_3_V_2_(PO_4_)_2_F_3_ is based on the one reported by Bianchini *et al.*^12,14,19^ This structure was modified to account for the full range of Na concentrations: Na_0_, Na_1_, Na_2_, Na_3_, and Na_4_, containing 0, 4, 12, and 16 Na atoms per unit cell, respectively. Every cell furthermore contains 8 atoms of P, 32 atoms of O, 32 atoms of F, 8 of V. Further configurations were generated based on our previous work, or available literature.^19,25,28^ For Na_0_ and Na_4_, only a single structure exists, either completely empty or completely filled with Na, respectively. Five structures were created for Na_1_ (Fig. S3), one of them being the structure experimentally solved by Bianchini *et al*.^13^ Four initial configurations were generated for Na_2_ as illustrated in Fig. S4, and three for Na_3_, as gathered in Fig. S5.

Based on the most energetically stable configurations for each Na content, all subsequent models were substituted with the different transition metals. A supercell was not necessary as we had already a large cell that contains more than 60 atoms.

The crystallographic unit cell was optimized by relaxing the atomic positions and lattice parameters until the forces on all atoms were below EDIFFG = −0.01 eV Å^−1^. The energy of the SCF cycles was converged within EDIFF = 10^−5^ eV. Fermi smearing with *σ* = 0.001 eV was required to ensure accurate energy convergence and to minimize electronic broadening, preserving the intrinsic bandgap and density of states distribution. To ensure valid results, the pseudopotentials were benchmarked to determine the most efficient ones, resulting in the selection of the following potentials: Na_pv for Na, _pv for Ti, V, Cr, Mn, standard potentials for Co, Fe, and Ni, and _sv for Mo, Nb, and Zr. An energy cutoff of 680 eV was used to ensure convergence.^36^ All calculations of the unit cell were performed using a *Γ*-centered *k*-point grid of 3 × 3 × 3 for structural relaxations.^37^ For Na bulk calculations, a first-order Methfessel–Paxton smearing with *σ* = 0.2 eV was applied to account for its metallic nature and ensure smooth electron occupation near the Fermi level, while a *k*-point mesh of 12 × 12 × 12 was used. The density of states (DOS) calculations were performed using a self-consistent field approach with ICHARG set to 1, ensuring that the charge density was updated during the self-consistent cycle to enhance accuracy. Furthermore, a denser *k*-point mesh (6 × 6 × 6) was employed and NEDOS was set to 5000 to provide a sufficiently fine resolution for DOS and pDOS (partial density of state). An ISMEAR value of −5 was set only for the DOS calculations. These calculations were processed using the Tool VASPKIT1.3.5 ^38^ to create the files of total and partial density of states and processed using OriginLab.

To investigate diffusion in the Na_*x*_M_2_(PO_4_)_2_F_3_-type structure, the transition states of the sodium diffusion pathways were investigated using the Nudged Elastic Band (NEB) method with climbing image convention.^39–43^ The initial and final images of each diffusion pathway were constructed based on the geometry of the optimized structures. These images were generated using the VTST tool package.^42,43^ The convergence for NEB is set to 0.05 eV Å^−1^. All obtained DFT total energies were in approximation treated as enthalpies at 0 K and calculated based on reported ICSD structures (Table S1). Their differences are used to estimate the Gibbs free energy of reaction at different temperatures using the following equation:

1 Δ_r_*G*(*T*) = Δ_r_*H*(0 K) − *T*Δ_r_*S*(*T*)

where Δ_r_*G*(*T*) represents the reaction Gibbs free energy at temperature *T*, Δ_r_*H*(0 K) is the enthalpy change at 0 K, and Δ_r_*S*(*T*) is the entropy contribution. For entropy contributions, we neglected the entropy of all solid reactants and products, assuming their contributions to be negligible compared to gaseous species. The temperature-dependent entropies for the gaseous species were obtained from the NIST-JANAF tables.^35^ The Gibbs free energies were evaluated at 0, 298.15, 400, 500, 700, 1000, and 1500 K to assess thermodynamic stability across different synthesis conditions. To compute the energies of the gaseous species, NH_3_, H_2_O, O_2_, and H_2_, a sufficiently sized box was created for each gas molecule to avoid interactions between periodic images. In addition, phonon calculations were carried out for the precursors and the products of the synthesis routes of Mn, Ti and Fe using finite displacement method as implemented in *phonopy*.^44^ The electronic convergence criterion was set to EDIFF = 10^−7^ eV and forces to EDIFFG = −0.003 eV Å^−1^.

All the figures presented in this paper were created using OriginLab for analysis and graphical representation, while structural visualizations were created using VESTA^45^ and Inkscape.^46^

## 3 Results and discussion

### 3.1 Na_*x*_V_2_(PO_4_)_2_F_3−2*y*_O_2_*_y_*

**Fig. 1:**
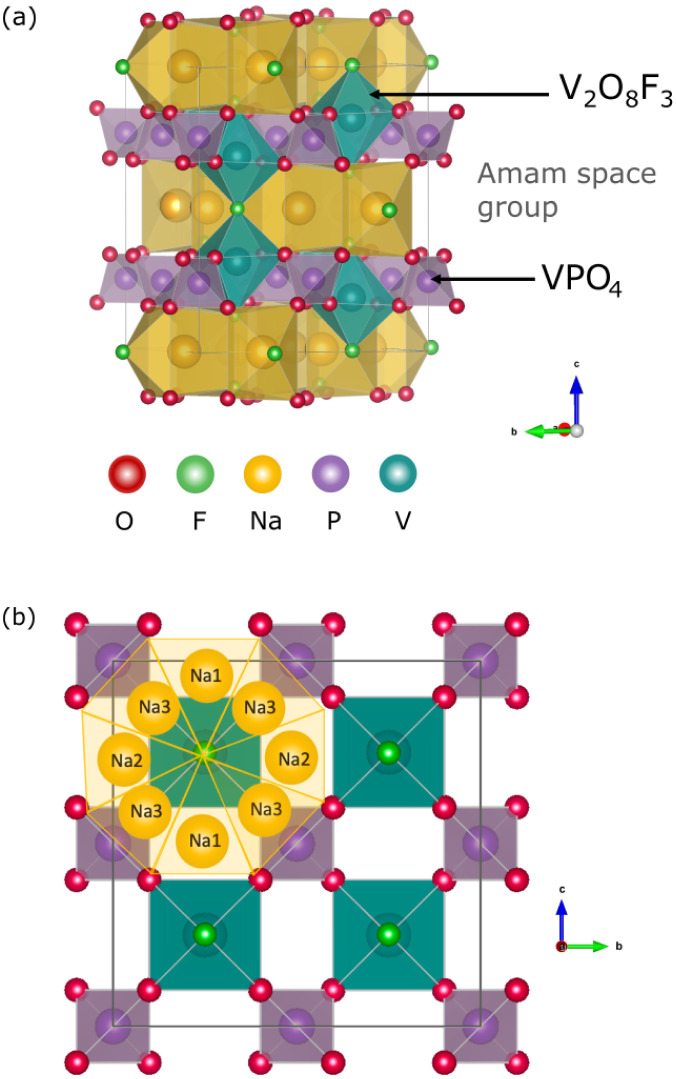
(a) Crystalline structure of Na_3_V_2_(PO_4_)_2_F_3_ indexed in space group *Amam*. The oxygen atoms are shown in red, fluorine in light green, sodium in yellow, phosphor in purple, and vanadium in cyan. (b) Arrangement of Na^+^ ions in the *ab*-plane of Na_3_V_2_(PO_4_)_2_F_3_, showing the presence of a ring-like configuration.

In the Na_*x*_V_2_(PO_4_)_2_F_3_ structure, V_2_O_8_F_3_ bi-octahedra form the primary framework, in which vanadium is coordinated by oxygen atoms with fluorine atoms occupying the positions along the bi-octahedra's long axis (Fig. 1a). These bi-octahedra are interlinked by PO_4_ tetrahedra, resulting in a three-dimensional framework with interstitial sites accessible to sodiation,^12,14,25,28^ forming two-dimensional Na layers within the *ab*-plane (Fig. 1b).

**Fig. 2:**
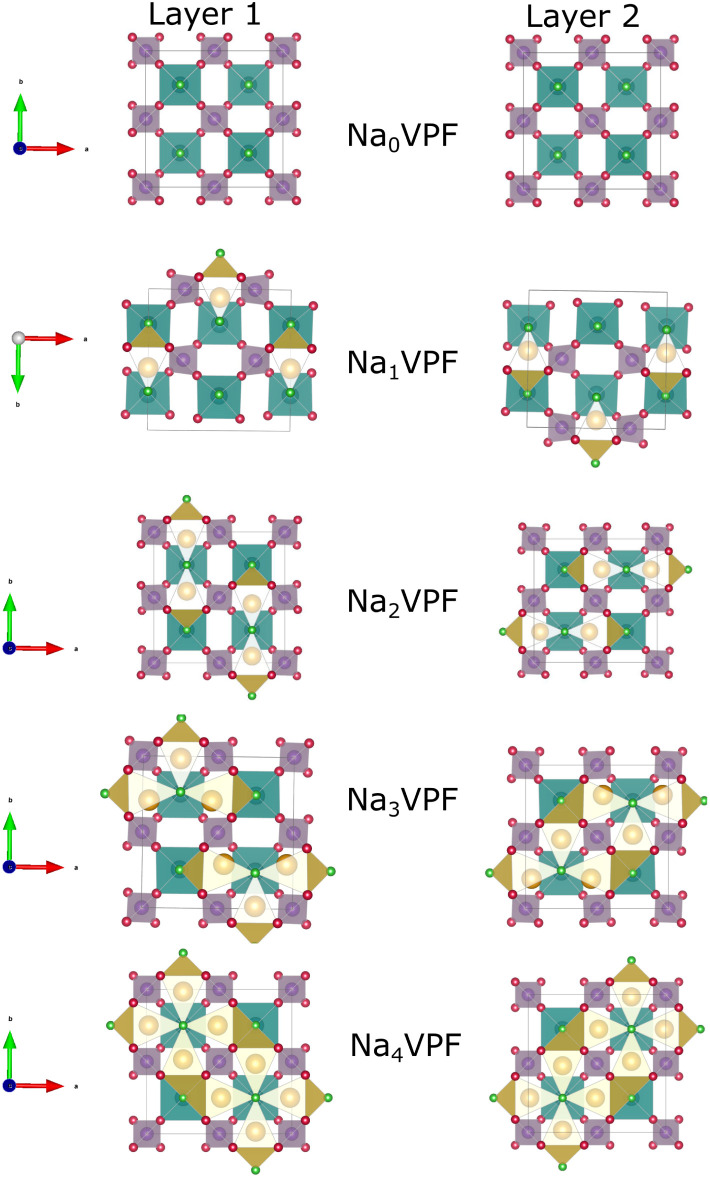
Ground state configurations of Na_*x*_V_2_(PO_4_)_2_F_3_ (0 ≤ *x* ≤ 4) showing the structural arrangement of Na ions in layer 1 (*z* = 0) and layer 2 (*z* = 1/2). These ground states, determined from the convex hull, represent the most stable configurations at each Na content and were used to compute the voltage profile of Na_*x*_V_2_(PO_4_)_2_F_3_.

**Fig. 3:**
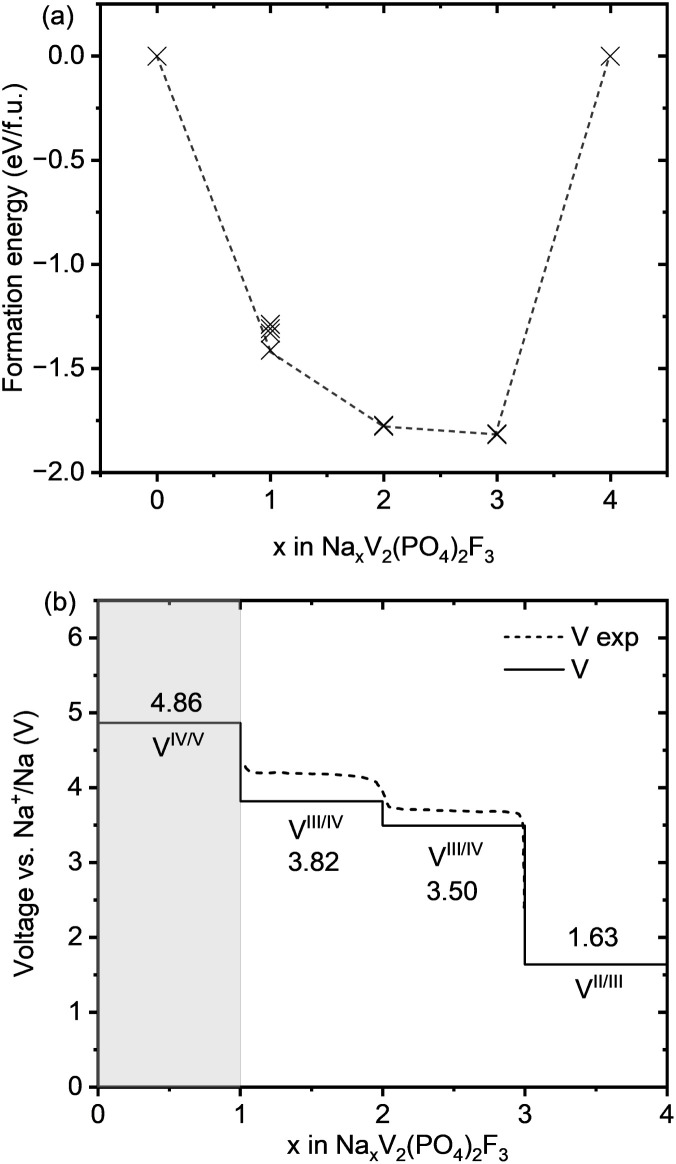
(a) Formation energies of various configurations of Na_*x*_V_2_(PO_4_)_2_F_3_ from *x* = 0 to *x* = 4, with the ground states represented by the dashed black line forming the convex hull. (b) Computed voltage profile of Na_*x*_V_2_(PO_4_)_2_F_3_ for Na contents from 0 to 4 (black line), compared to the experimental data (black dashed line) taken from Akhtar *et al*.^19^ The light gray-shaded area indicates Na concentration ranges in which Na ions are likely not accessible, based on typical values of the electrochemical electrolyte stability window (1–4.7 V *vs.* Na^+^/Na).^20,25,26^

Throughout this manuscript, chemical formulae are abbreviated for simplicity: Na_*x*_V_2_(PO_4_)_2_F_3_ is referred to as Na_*x*_VPF and Na_*x*_M_2_(PO_4_)_2_F_3_ is abbreviated as Na_*x*_MPF. M may be any element from the transition metals mentioned in the methods section, and the sodiation level *x* is specified by the subscript, *e.g.*, Na_1_M_2_(PO_4_)_2_F_3_ is written as Na_1_MPF. Na_3_VPF adopts an orthorhombic structure in the space group *Amam*. It contains three distinct sodium positions that are arranged in a circle around the fluorine centers. The sites are indexed as Na(1) (4*c*) and Na(2) (8*f*), which are capped trigonal-prismatic sites along the [010] and [100] directions from the center of the bi-octahedra, and Na(3) (8*f*), which are smaller trigonal-prismatic sites along the [110] direction, respectively, for a total of 8 Na sites per ring.^25^ Given their vicinity to each other, these Na(2) and Na(3) sites are partially occupied at approximately 70% and 30% for Na_3_VPF, respectively. This indicates that the larger Na(1) and Na(2) sites are more stable compared to Na(3).^11–13^ While the partial occupancy of Na(2) and Na(3) sites is important in modulating Na^+^ diffusion paths, computations demand fully occupied Na sites. Accordingly, three configurations were manually created for Na_3_VPF (Fig. S4). The most stable configurations at each Na concentration are shown in Fig. 2. The configurations for all other sodium ratios were created based on previous studies^13,25,28^ and our work.^19^ All the ground state structures of Na_*x*_VPF 0 ≤ *x* ≤ 4 are presented in the Fig. 2, and the energies obtained from these are then used to construct a convex hull, as reported in Fig. 3a.

The formation energies were calculated using the equation below,^28^ where the pV-term and the entropic contributions are neglected:2
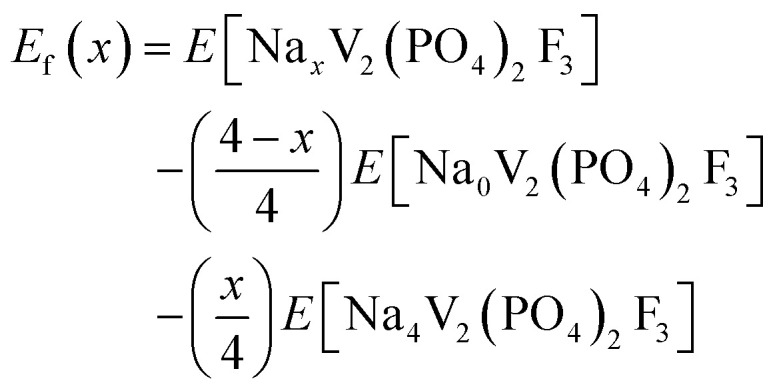


These formation energies construct a convex hull for the Na_*x*_VPF system (0 ≤ *x* ≤ 4). Such convex hull depicts the most stable configurations, while the configurations above the convex hull are classified as metastable, indicating that they may decompose into neighboring ground-state phases. Among the most stable structures, Na_3_VPF has the lowest formation energy, while configurations Na_1_VPF and Na_2_VPF show slightly higher formation energies. For Na_2_VPF and Na_3_VPF, the energetic spread of the investigated configurations is approximately 5 meV f.u.^−1^ and 4 meV f.u.^−1^, respectively, so not visible in Fig. 3. In the Na_1_VPF structure reported by Bianchini *et al.*^13^ with *Cmc*2_1_ as the space group, sodium occupies only Na(1) sites. Among the configurations investigated, the most stable one for Na_1_VPF matches the one experimentally reported.^13^ The voltage profile for V (Fig. 3b) and all substitutions was calculated from the convex hull based on the ground state structures shown in Fig. 2a. The individual voltages were computed according to:^28^3
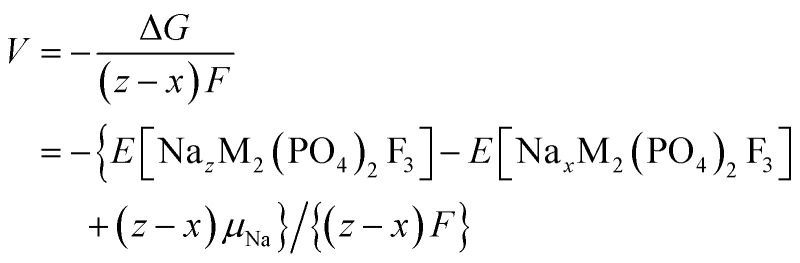
where M is transition metal, *μ*_Na_ is the chemical potential of bulk Na and F is the Faraday constant. The light gray-shaded area in Fig. 3b indicates which Na concentrations are not accessible during reversible cycling, based on typical values of the electrochemical electrolyte stability window (1–4.7 V *vs.* Na^+^/Na).^20,25,26^ Our computed voltages match the available experimental voltage profiles for Na_*x*_VPF.^12,14,19,26,47,48^ The first voltage plateau from Na_3_VPF to Na_2_VPF is found at 3.50 V (*vs.* 3.6 V experimental), while the second one from Na_2_VPF to Na_1_VPF lies at 3.82 V (*vs.* 4.1 V experimental), both of which rely on the redox couple V^3+^/V^4+^. The low voltage plateau to reach Na_4_ from Na_3_, the fully sodiated structure, shows a rather low voltage of 1.63 V, which is close to the experimental values.^14^ Finally, the extraction of the third sodium from Na_1_VPF to VPF is calculated to occur at 4.86 V, while it was previously reported at 4.9 V.^47^ Our findings underestimate the experimental results by ∼0.10 to 0.28 V for the range 1 ≤ *x* ≤ 3.^19^ It is likely that the extraction of the third Na ion occurs at a voltage higher than the computed value, which explains why, with the exception of few reports,^22,25,28^ this is usually not observed. Previous studies that employed GGA + U^28^ observed the voltage plateaus for 0 ≤ *x* ≤ 4 at 4.53 V, 3.60 V, 3.24 V and 1.44 V, respectively, which underestimates the experimental findings more than r^2^SCAN-D4 + U.

The DOS and pDOS for Na_*x*_VPF are reported in the SI (Fig. S6), showing the process of activation of the V^4+^/V^5+^, V^3+^/V^4+^ and V^2+^/V^3+^ redox couples, respectively. In the fully sodiated structure Na_4_VPF, the DOS exhibits a peak split around the Fermi level, indicative of the coexistence of V^2+^/V^3+^. Upon partial desodiation to Na_3_VPF, additional unoccupied states emerge just above the Fermi level, suggesting the full oxidation of V^2+^ to V^3+^. In Na_2_VPF, the presence of sharp and localized peaks at the Fermi level supports the presence of the V^3+^/V^4+^ redox couples. In Na_1_VPF, broadening of the electronic states near the Fermi level indicates delocalization, which aligns with the full oxidation to V^4+^. Finally, in the fully desodiated Na_0_VPF, a distinct splitting of electronic states at the Fermi level accompanied by the appearance of additional peaks confirms again the coexistence of V^4+^ and V^5+^ oxidation states.

### 3.2 Substituted phosphate fluorides

**Fig. 4:**
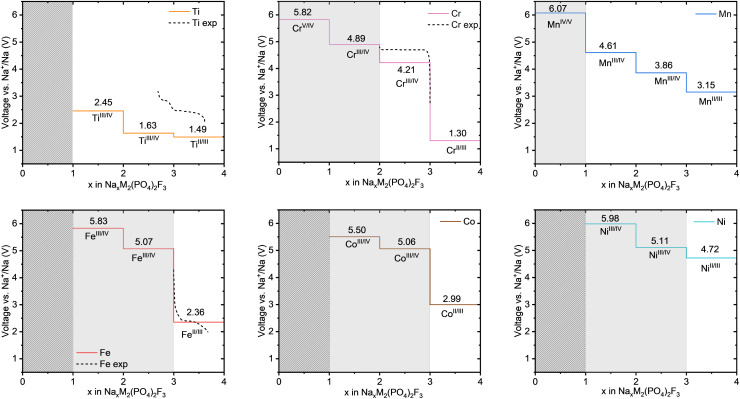
Voltage-composition curves for Na_*x*_M_2_(PO_4_)_2_F_3_ (0 ≤ *x* ≤ 4), showing the computed voltages for first row transition metals M = Fe, Cr, Ti, Mn, Ni, Co. The light gray region indicates voltages outside the stability window of most electrolytes, while the dark gray shaded region represents inaccessible ranges where oxidation beyond M^4+^ is not achieved. Experimental values for Ti, Fe, and Cr are included for comparison (black dashed lines), as gathered from.^27,49,50^

Having benchmarked the V-based compound, we now proceed to substitute V in Na_*x*_VPF with various 3d and 4d transition metals (Co, Cr, Fe, Mn, Ni, Ti, Mo, Zr and Nb) to evaluate the operational voltages of the resulting structures. Fig. 4 illustrates the voltage profiles for Na_*x*_MPF in the range of 0 ≤ *x* ≤ 4 with the investigated 3d transition metals, where distinct voltage plateaus are observed from the fully desodiated MPF to the fully sodiated Na_4_MPF. Each transition metal exhibits different voltage plateaus depending on the redox couple involved and on the electronic energy of the bands involved in such redox reactions. Co, Fe, Ti, and Ni are only expected to work in the range 1 ≤ *x* ≤ 4, *i.e.*, for oxidation states from M^2+^ to M^4+^, and not in the range 0 ≤ *x* ≤ 1 (dark gray shaded region in Fig. 4). In contrast, Cr and Mn can reach the redox couple M^4+^/M^5+^, even if that is found to require a very high voltage above the common electrolytes stability window with 5.82 V and 6.07 V, respectively (light gray region in Fig. 4). From an application standpoint, although some computed voltages exceed the typical electrolytes stability window, suitable high-voltage Na electrolytes may provide alternative solutions: ionic liquids can reach to 4.8–5.0 V *vs.* Na^+^/Na,^51,52^ while high concentration ether electrolytes^48^ and quasi-solid/solid electrolytes may even exceed 5 V *vs.* Na^+^/Na.^53,54^ Co, Cr, Fe and Ni all exhibit a too high voltage above 5 V in the compositional range 3 ≤ *x* ≤ 1. For Cr and Co, which have half-filled and completely filled *t*_2g_ electronic levels,^50^ the high voltage comes from removing electrons from these stable levels lying at low energy as is shown in the DOS reported in Fig. S10 and S13. The DOS for Co also shows clearly its preference for low spin configurations. On the contrary, for Fe the high voltage originates from the fact that the transition metal bands lie too low in energy, so further oxidation would occur *via* electronic states associated to the oxygen bands, as can be seen by the lack of transition metal pDOS contribution to the total DOS near the Fermi level (Fig. S12). Here, the high stability of the high-spin d^5^ configuration prevents further oxidation beyond Fe^3+^. In the case of Mn, a similar situation prevents further oxidation beyond Mn^4+^ (Fig. S11) due to the high stability of the *t*_2g_ orbital. On the other hand, the Mn^3+^/Mn^4+^ redox couple can be accessed resulting in reasonable voltages of 3.86 V and 4.61 V. Likewise, a voltage plateau of 4.21 V was calculated for Cr, which is relatively close to the experimental value, but underestimated by ∼0.5 V,^50^ yet still accessible experimentally. Ti^3+^/Ti^4+^ exhibits a plateau at 1.63 V, which is at a substantial discrepancy of ∼1.07 V to the experimental value of 2.7 V during Na (de)intercalation.^49^ These discrepancies (either for Ti or Cr) may arise from Hubbard U correction, which can overlocalize d-electrons, and from the influence of Cr^3+^ and Ti^3+^ magnetic interactions.^55–57^ In addition, it can be related to the transferability of U values. These are calibrated on binary oxides but are not universal for all our mixed anion O/F materials, since U values are system and environment dependent.^58^ Moreover, residual self-interaction error under *meta*-GGAs functionals can occur even when using U, which leaves early 3d d^1^/d^2^ slightly under-localized.^34,59,60^ Ultimately, it is important to keep in mind these potential offsets when considering the suitability of substituted materials as positive or negative electrodes.

Considering an electrochemical discharge of the pristine samples (*i.e.* the range 4 ≤ *x* ≤ 3), the redox couples Ti^2+^/Ti^3+^ and Cr^2+^/Cr^3+^ for Ti and Cr are accessible for reversible cycling but with very low voltages, however, the experimental value for Ti is underestimated by 0.91 V. Co and Mn with redox couples Co^2+^/Co^3+^ and Mn^2+^/Mn^3+^ offer more promising voltage values of 2.99 V and 3.15 V, respectively. Fe (Fe^2+^/Fe^3+^) also exhibits a voltage plateau of 2.36 V, which is well in line with the experimental value of 2.45 V measured by our group^27^ and others.^61^ The reported voltages are strongly linked to the energetic position of the transition metal *t*_2g_ and 
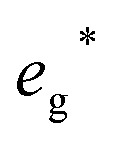
 orbitals in the materials, which can be seen in the DOS for V, Cr, Mn, Fe, Co, and Mo in the SI (Fig. S6, S10–S14). In conclusion, most investigated cations have redox couples which exhibit a potential that is too high for stable operation over a range of at least 2 Na ions exchanged. Mn represents a notable exception, since 3 Na ions appear to be at excellent voltages for electrochemical operation. While Na_3_MnPF has never been reported experimentally, if it could be stabilized, it would yield a specific energy of 693.97 Wh kg^−1^ for the range 4 ≤ *x* ≤ 1.

**Fig. 5:**
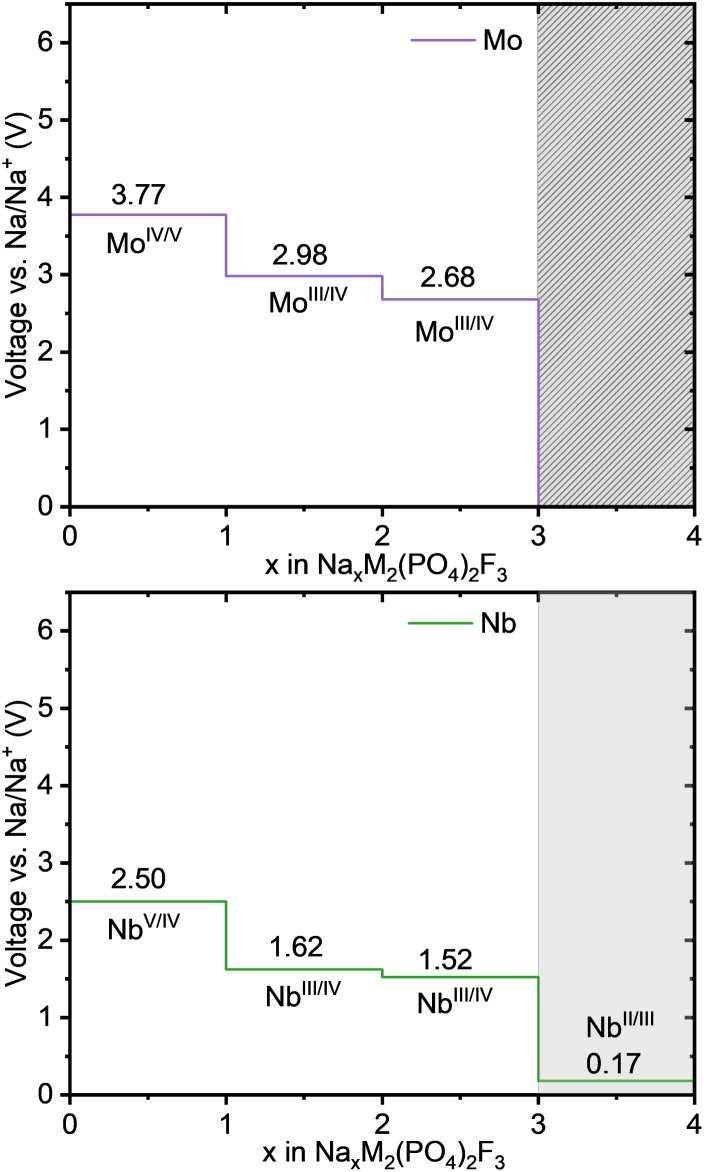
Voltage-composition curves for Na_*x*_M_2_(PO_4_)_2_F_3_ (0 ≤ *x* ≤ 4), showing the computed voltages for selected second row transition metals M = Mo, Nb. The light gray region indicates voltages outside the stability window of most electrolytes, while the dark gray shaded region represents inaccessible ranges where oxidation states below Mo^3+^ are not achieved.

Subsequently, the three 4d transition metals Zr, Nb, and Mo were considered for substitution. The voltage values obtained for Nb and Mo are reported in Fig. 5. The results for Zr are to be found in the SI (Fig. S7–S9), but are not considered further as it only yields very low voltage values between 0.6 and 0.06 V. The voltage profiles of Nb and Mo are below their 3d counterparts. Nb shows plateaus at 0.17 V, 1.62 V, 1.52 and 2.50 V corresponding to the redox couples Nb^2+^/Nb^3+^, Nb^3+^/Nb^4+^ and Nb^4+^/Nb^5+^, which is 1.46–2.36 V below the corresponding redox couples of V. Our findings are in agreement with the results by Van der Lubbe *et al.*,^28^ who reported 0.28 V, 1.38 V, 1.72 V, and 2.51 V, respectively. Van der Lubbe *et al.* reported that extracting the third sodium ion in Nb-based cathode would offer an additional 20% capacity (160 mA h g^−1^) with respect to the original V-based material (128 mA h g^−1^), but the lower voltage plateaus would reduce the energy density by 40.6%.^28^ Similarly, Mo demonstrates plateaus at 2.68 V, 2.98 V and 3.77 V related to the redox couples Mo^3+^/Mo^4+^ and Mo^4+^/Mo^5+^ confirmed by the DOS (reported in Fig. S14). These values lie 1.53–2.05 V below the corresponding redox couples of Cr. Na_4_MoPF is not shown because no stable structure with Mo^2+^ could be obtained by r^2^SCAN, reflecting the fact that it is not a stable oxidation state for molybdenum. Using GGA + U, Mo^2+^/Mo^3+^ had been observed at an extremely low voltage of 0.18 V.^28^ Nevertheless, molybdenum also appears as a promising cation in the NaMPF framework, as the three remaining voltage plateaus lie well within the regular electrolyte stability window.

### 3.3 Crystal structures

**Fig. 6:**
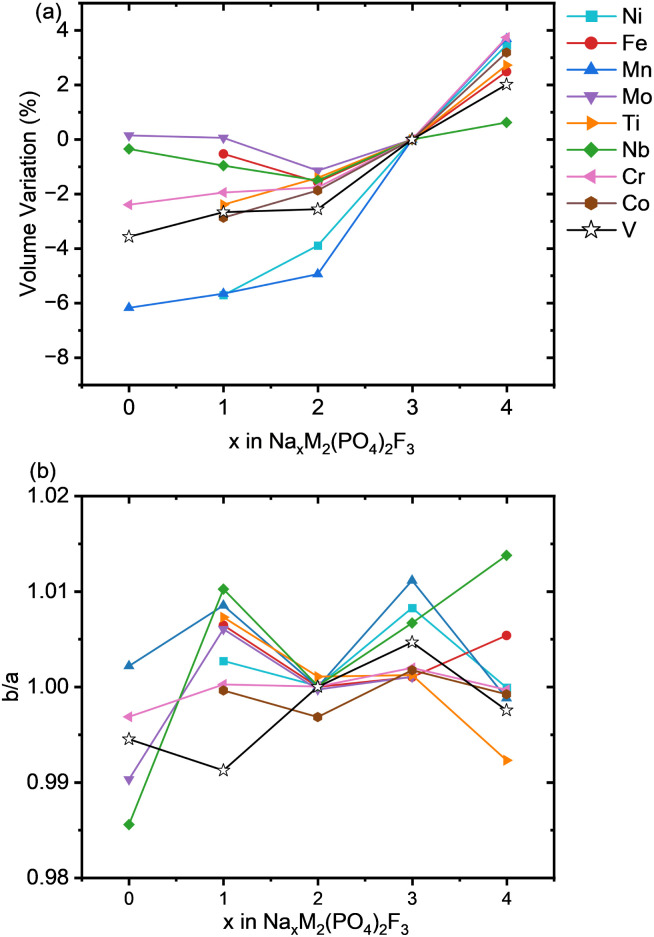
(a) Volume variation as a function of Na content in Na_*x*_M_2_(PO_4_)_2_F_3_ (0 ≤ *x* ≤ 4) for various 3d and 4d transitions metals, (b) *b*/*a* ratio evolution illustrating the orthorhombic *vs.* tetragonal nature of the ground state structures as a function of Na insertion and extraction.

After the voltages in Na half cells, the structural characteristics of the substituted phosphate fluorides were investigated. Fig. 6a illustrates the volume change and Fig. 6b the *b*/*a* ratio for Na_*x*_M_2_(PO_4_)_2_F_3_ cathodes as function of Na content *x*. All M−O and M−F distances are gathered in the SI (Fig. from S15 to S23), providing information in particular about the different M−O and M–F bond lengths obtained. The relative volume variation displayed in Fig. 6a for all investigated structures is normalized to the volume of the Na_3_MPF compounds. Overall, we observe a clear reduction of unit cell volume with decreasing Na content for most substitutions from *x* = 3 to *x* = 2, which is in line with the decreasing size of transition metals when oxidized. However, for sodium concentrations *x* ≤ 2, the overall volume remains similar across most transition metals. Generally, Ni and Mn based compounds exhibit the largest volume variation at −4% from *x* = 3 to *x* = 2, which can be related to the particularly large relative variation of ionic size in these cations (from 0.645 Å  to 0.53 Å  and from 0.56 Å  to 0.48 Å  in the case of Mn and Ni respectively). All other investigated transition metals exhibit a smaller deviation of only −1 to −2.5% from *x* = 3 to *x* = 2, with Mo, Nb, and Fe changing the least. For Mo and Nb, this is due to the larger, more polarizable cations, and for Fe, it is due to the metal ion retaining its oxidation state, as indicated by the corresponding pDOS (Fig. S9). Upon further oxidation to Na_1_MPF, the volume of the compounds M = Cr, V, and Ti diminishes by approximately −3% indicating a similar structural response upon Na extraction. In contrast, Nb, Fe and Mo still show minimal volume change, with variation ranging from 0 to ∼−1%. Notably, Mn and Ni display the highest volume reduction at −6%. For the fully desodiated compounds, the structure is entirely governed by the intrinsic characteristics of the transition metal cations and the interconnecting framework of anions, as no Na ions are present. The MPF structure with Mn exhibits the largest total volume reduction of −6%, followed by V and Cr (−3.5% and −2% respectively), while the structures with Mo and Nb remain nearly unchanged (0.5%). This can be explained by the fact that 4d metals Mo and Nb, as compared to 3d ones, have more closed electronic shells screening the valence d-orbitals, hence weaker electrostatic interactions and a larger amount of covalent bonding ultimately resulting in lower size variations.^62^ The shorter M–O and M–F bond lengths observed for Mn, Cr, and V further confirm their stronger ionic bonding nature. Conversely, Mo and Nb exhibit longer bonds, indicating their greater covalency and weaker electrostatic attraction to oxygen and fluorine (Fig. S15, S17, and S19 respectively). Considering finally the sodiation (to *x* = 4), the volume is the greatest for all transition metals, as expected by the larger Na concentration and the increasing ionic size of cations with lower valence.^63^ For Mn, Mo, and Cr, a significant expansion by 4% is observed, and they exhibit longer bonds with oxygen and fluorine due to their lower oxidation state (Fig. S17 to S19). V, Co, Fe, and Ti undergo a more moderate expansion of ∼2%, indicating a balance between the Na insertion effects and the structural resilience of their frameworks. This is in good agreement with experimental results of the sodiation of Na_3_VPF towards Na_4_VPF.^14^

Fig. 6b illustrates the *b*/*a* ratio as function of Na content, showing the shift in symmetry during the insertion and extraction of Na, specifically providing insight into the change between an orthorhombic and a tetragonal symmetry. In fact, Na_3_VPF was originally believed to be tetragonal, before the small orthorhombic distortion could be recognized.^12^ However, other compounds such as Na_3_FePF are still reported as tetragonal,^49^ or having even smaller orthorhombic distortions.^27^ In good agreement with this, we find that at *x* = 3 the compounds based on Fe, Ti, Cr and Mo have a ratio *b*/*a* of essentially 1, *i.e.*, they are expected to remain tetragonal. On the other hand, Na_3_VPF is correctly identified as orthorhombic, with a *b*/*a* close to 1.005. The other compounds are all found to be orthorhombic with even larger deviations from the tetragonal symmetry with respect to Na_3_VPF. The largest distortion is observed for Nb and Mn. The Mn system (d^4^) shows a clear splitting of the Mn–O bonds, where two bonds are considerably shorter than the others, as illustrated in the Fig. S17.

As the materials are desodiated to *x* = 2, we observe a tendency for all structures to become tetragonal (with the exception of Co and Ti, which maintain a very slight distortion). This is reasonable as in Na_2_VPF the distribution of Na ions is isotropic (collinear arrangement) along the *a* and *b* axis in the two *ab* planes at *z* = 0 and *z* = 1/2. On the other hand, at *x* = 1 a pronounced elongation of the *b*-axis is observed for all the materials. This elongation, which can be noticed also from the M–O and M–F bond lenghts in the SI (Fig. S15–S23), is likely related to the sodium arrangement. For *x* = 0, where no Na is present, the unit cell is orthorhombic for most transition metals that can reach this oxidation state. As no Na is present, here the distortion must be related to the presence of two cations in the unit cell with different sizes leading to different bond lenghts and especially different M-F-M torsional angles in the bioctahedra.

### 3.4 Na migration barriers

**Fig. 7:**
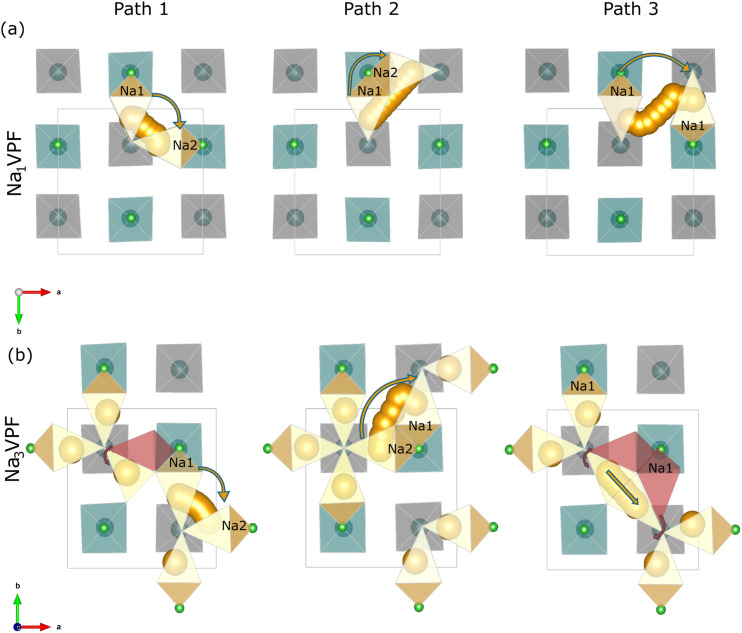
Top-down (*ab*-plane) view of Na ion migration pathways in Na_1_VPF (a) and Na_3_VPF (b), highlighting Path 1 (intra-unit migration), Path 2 (inter-unit migration between Na1 and Na2 sites), and Path 3 (inter-unit migration between Na1 sites). The red-colored octahedra represent the initial positions of Na ions before relaxation, showing the site preference shift upon structural relaxation. The Na migration pathways are specified by orange arrows. The green octahedra represent those with fluorine atoms (in bright green) in the same plane as the Na ions (at *z* = 0), while the grey ones correspond to those higher along the *c* axis (apical fluorine at *z* = 0.127, not shown).

Nudged elastic band (NEB) simulations were carried out to calculate the migration barriers for Na_*x*_VPF (*x* = 0, 1, 3) as well as for selected doped compounds, *i.e.*, Na_*x*_FePF, Na_*x*_MnPF, and Na_*x*_MoPF (*x* = 1, 3), elucidating the effects of Na content and transition metal substitution. For the high-vacancy limit, both Na_0_VPF and Na_1_VPF were investigated. The former represents a fully deintercalated system, containing only one Na ion per unit cell to simulate the migration pathway, and was chosen to better compare with existing literature. The latter represents a partially sodiated state (Fig. 7) with introduction of one Na vacancy. For the discharged system with only few vacancies (low vacancy limit), a pristine structure of Na_3_VPF was used where a Na atom was removed.

Fig. 7a and S24 indicate the three migration pathways considered at the two high-vacancy sodium concentrations, respectively. Fig. 7b exhibits instead the pathway for the high-Na concentration (*x* = 3). As can be seen, the NEB calculations target three types of migration pathways. Path 1 corresponds to the hopping of sodium ions between adjacent Na1 and Na2 sites at the same fluorine anion *via* the interstitial site Na3, forming a localized diffusion channel (which we can name intra-unit, because Na ions form a ring above a given bioctahedral unit). Path 2 and Path 3 instead allow Na migration between sites that are bonded to different fluorine anions (inter-unit, as these paths link different bioctahedral units). Path 2 links a Na1 site with a Na2 site across two Na3 interstitial sites. Path 3 links a Na1 site with another Na1 site *via* two Na3 interstitial sites. It can be intuitively understood that only a local Na hopping is involved in Path 1, while long range (percolating) Na diffusion require either hops along Path 2 or along Path 3.

To enable accurate computational modeling of Na-ion migration pathways, slight modifications to the nominal Na composition were necessary. Specifically, to investigate Na_0_VPF (Fig. S24), one Na atom was added to the structure, resulting in an effective composition of Na_0.25_VPF. Conversely, for Na_1_VPF (Fig. 7a), one Na atom was removed, corresponding to an investigation of Na_0.75_VPF. Similarly, in the case of Na_3_VPF (Fig. 7b), the removal of one atom from one of the rings corresponds practically to investigating composition Na_2.75_VPF.

The *ab*-plane view for Na_*x*_VPF is shown in Fig. 7, with the three crystallographically distinct Na sites Na1, Na2, and Na3 as displayed in Fig. 1b. However, structural relaxation of Na_3_VPF reveals significant site preference shift. Instead of only occupying Na1 and Na2 sites (red octahedra in Fig. 7), Na also occupies Na3 sites to minimize electrostatic repulsion with its neighboring cations. This rearrangement is expected to decrease the energies barriers for this structure, specifically for path 3 due to closer starting and end states.

**Fig. 8:**
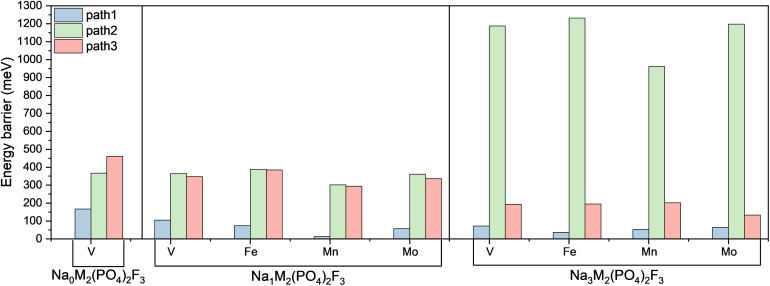
Histogram illustrating the energy barriers for Na ion migration along three different pathways in Na_0_MPF, Na_1_MPF and Na_3_MPF with M = V, Fe, Mn, and Mo. The pathways shown are: Path 1 corresponds to intra-unit migration, Path 2 to inter-unit migration between Na1 and Na2 sites, and Path 3 to inter-unit migration between Na1 sites. The values plotted here are summarized in Table 1.

As summarized in Table 1 and illustrated in Fig. 8, the computed migration barriers for local intra-unit migration (Path 1) are significantly lower than inter-unit exchange between sites bound to different fluoride anions (Paths 2 and 3). This confirms the expectation that Na diffusion is easier within local coordination units than across different bioctahedral units, and that adjacent Na1 and Na2 sites readily exchange ions and vacancies *via* Na3 sites. The substitution of V with different transition metals does not substantially alter Path 1 Na migration barriers, which remain consistently very low. On the other hand, much higher barriers are observed for Paths 2 and 3.

**Table 1:** Migration barriers for Na_0_V_2_(PO_4_)_2_F_3_, Na_1_M_2_(PO_4_)_2_F_3_, and Na_3_M_2_(PO_4_)_2_F_3_ for M = V, Fe, Mn and Mo

Material	Transition metal	Path 1 (meV)	Path 2 (meV)	Path 3 (meV)
Na_0_M_2_(PO_4_)_2_F_3_	V	167	367	461
Na_1_M_2_(PO_4_)_2_F_3_	V	105	365	347
	Fe	74	388	385
	Mn	13	302	294
	Mo	57	361	336
Na_3_M_2_(PO_4_)_2_F_3_	V	72	1188	193
	Fe	38	1232	195
	Mn	53	962	202
	Mo	64	1198	133

NEB calculations were performed for Na_0_VPF, as this composition has been investigated in previous studies, to enable a direct comparison with present results. The migration barriers for Na_0_VPF are 167, 367, and 461 meV for Paths 1, 2, and 3, respectively, exceeding the corresponding GGA + U values of 134, 300, and 290 meV reported by Van der Lubbe *et al.*^28^ and Dacek *et al.*^25^ This systematic increase suggests that r^2^SCAN may improve treatment of exchange-correlation and dispersion effects to capture subtle energetic contributions more accurately.

In Na_1_MPF (M = Fe) the barrier for Path 1 is reduced from 105 meV to 74 meV. Even lower barriers are observed for Mn and Mo (13 meV and 57 meV, respectively). For Paths 2 and 3, Fe exhibits a mixed trend. While it lowers the intra-unit barrier (Path 1), it increases the inter-unit barrier by 23 meV and 38 meV for Path 2 and Path 3, respectively. In contrast, Mn and Mo exhibit reduced energy barriers for all paths compared to vanadium.

To the best of our knowledge, while the Na-ion migration pathways in Na_0_VPF and Na_4_VPF have been reported earlier,^25,28^ those in Na_3_VPF have not been investigated in any previous studies using NEB. Therefore, we focus on it in this work to complete the picture on the ion conduction properties of Na_*x*_VPF.

In the Na_3_MPF structures, for path 1, the barrier drops from 72 meV (V-Based) to 38 meV for Na_3_FePF, similarly to Mn and Mo. Moving to path 2, Mn is reducing the energy barrier compared to Fe and Mo that increased the barrier by 226 meV. Fe and V show similar migration barriers for path 3 (195 meV and 193 meV, respectively), while Mn exhibits slightly higher values, and Mo consistently reduces the barrier. The increase in migration barriers for inter-unit pathways in the low-vacancy limit is attributed to stronger electrostatic repulsions among Na ions. Conversely in high vacancy limit, Na ion migration benefits from reduced electrostatic interactions, resulting in lower barriers.

Overall, our results confirm that while Na migration along path 1 is very fast, the rate-limiting step is always Path 2 or Path 3. In our calculations, Path 2 exhibits by far the highest barrier for pristine materials, but only one of these two paths is needed to achieve sodium percolation. Therefore, we can use Path 3 to estimate the rate capability of the different Na_*x*_MPF cathode materials. In general, across all materials the Path 3 limiting barrier is quite low but increases with decreasing Na content from values of the order of 133–202 meV to 294–385 meV. In the pristine state, Fe and Mn slightly slow down the kinetics as compared to vanadium, while Mo significantly enhances it. In the charged state (*x* = 1), Fe still has worse kinetics, while Mn and Mo both provide a slight advantage as compared to V. However the ground state structures with strong Na^+^/vacancy ordering impeding ion transport at stoichiometric compositions such as Na_1_V_2_(PO_4_)_2_F_3_ ^25^ are still stable in the substituted materials, indicating it may be kinetically challenging to reach Na contents below *x* = 1.

### 3.5 Synthesis routes

**Fig. 9:**
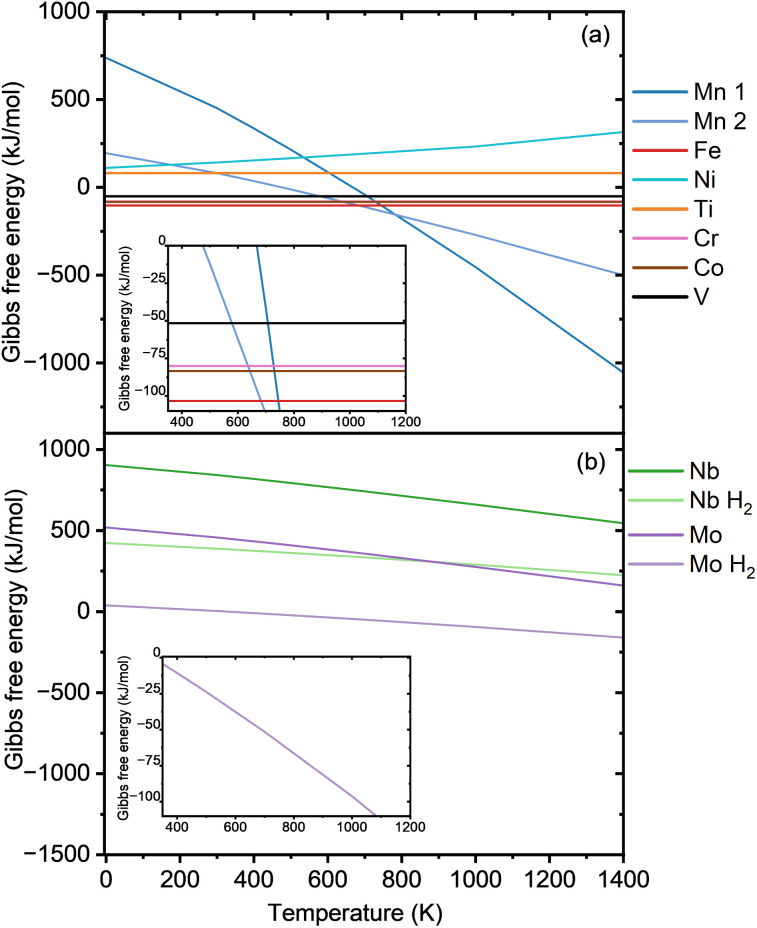
Gibbs free energy as a function of temperature for various Na_3_M_2_(PO_4_)_2_F_3_ compounds where M are transition metals from the first and second row, calculated based on the reactions equations detailed in Table 2.

Finally, possible synthetic routes for the substituted phosphate fluorides are investigated. We consider here only solid-state reactions, but it should be kept in mind that several examples of hydro- and solvothermal reactions are also reported in the literature for phosphate fluorides. In all cases the precursors considered are stable compounds reported in the ICSD (listed in Table S1). The main route is based on the experimentally proven synthesis of Na_3_V_2_(PO_4_)_2_F_3_, which uses the precursors VPO_4_ and NaF. Fig. 9 presents the Gibbs free energy as a function of temperature for Na_3_M_2_(PO_4_)_2_F_3_ based on the synthesis routes shown in Table 2, which includes additional reductive or oxidative conditions using O_2_ or H_2_. Only the entropy of the gases is considered, as obtained from the JANAF tables,^35^ for all the necessary gaseous precursors and products. All details of these calculations are given in the Methods section. For Fe-, Co-, Cr-, and Ti-based cathodes, where a stable trivalent phosphate MPO_4_ like VPO_4_ exists, the straightforward route using the trivalent phosphate precursors and NaF is investigated. Fig. 9a shows a negative Gibbs free reaction energy for M = Fe, Co, and Cr, suggesting that the corresponding NaMPF compounds can be synthesized. As no gaseous compounds participate in the proposed reaction, Δ*G* is not expected to be influenced by temperature. The synthesis of both the Fe- and Cr-based NaMPF compound has been proven experimentally.^27,50,61^ Two different routes are proposed for Mn because anhydrous MnPO_4_ is not a stable compound. The first uses Mn_2_O_3_ as Mn source and (NH_4_)H_2_PO_4_ as phosphate source, while the second is based on MnPO_4_·H_2_O. The Gibbs free energy (Fig. 9a) for Mn with the first route suggested (Mn 1) remains positive until a temperature around 667 K (393.85 °C), then it turns negative due to the entropy contribution of the released water and ammonia. For the second route (Mn 2), the Gibbs free energy turns negative at around 475 K (201.85 °C) at a shallower slope compared to the first Mn synthesis route due to the smaller stoichiometric amount of released gas. Here it should be mentioned that NH_3_ and H_2_O are released at ∼190–320 °C^64^ and ∼200–300 °C,^65^ respectively, so a temperature hold at low temperature in Ar or N_2_ may be helpful to eliminate these gases. For the synthesis of the Ni-based compound, the oxidation of Ni(ii) pyrophosphate is proposed, as shown in Fig. 9a and Table 2, but the reaction is not found to be thermodynamically favorable. Nb and Mo compounds can be synthezised from their respective stable oxyphosphates MOPO_4_. The reduction to the trivalent state can be achieved either with a calcination in an inert atmosphere (assuming O_2_ release), or active reduction with hydrogen. The reaction of NbOPO_4_ and NaF in Argon gives a consistently positive Gibbs free energy. Even for a reduction with hydrogen, as shown in the second route in Table 2, the Gibbs free reaction energy remains positive at all considered temperatures. Two analogous synthesis routes are proposed for Molybdenum. As observed in Fig. 9b, the synthesis route using MoOPO_4_ and H_2_ gas yields a negative Gibbs free energy (above 314 K), indicating that this may be a valid synthesis route to Na_3_Mo_2_(PO_4_)_2_F_3_. To confirm the robustness of the thermodynamic results despite having neglected the entropy of the solid phases, the vibrational entropies of solid precursors and products were included based on phonon calculations for selected materials, *i.e.,* Na_3_FePF, Na_3_MnPF, and Na_3_TiPF, as shown in Fig. S25. For the Mn routes (Fig. S25a), the Gibbs free energies remain negative across the studied temperature range, although with a reduced slope. For Ti (Fig. S25b), the Gibbs free energy increases with temperature, confirming that the reaction is thermodynamically unfavorable. For Fe (Fig. S25c), the Gibbs free energy becomes progressively more negative with temperature, although the variation is very small. These results indicate that the Gibbs free energy trends are robust even when neglecting the entropy of solids.

**Table 2:** Synthesis reactions for Na_3_M_2_(PO_4_)_2_F_3_ for various transitions metals to investigate the influence of reaction routes and the environment on thermodynamic stability

Element	Reaction
V	2VPO_4_ + 3NaF → Na_3_V_2_(PO_4_)_2_F_3_
Cr	2CrPO_4_ + 3NaF → Na_3_Cr_2_(PO_4_)_2_F_3_
Fe	2FePO_4_ + 3NaF → Na_3_Fe_2_(PO_4_)_2_F_3_
Ti	2TiPO_4_ + 3NaF → Na_3_Ti_2_(PO_4_)_2_F_3_
Co	2CoPO_4_ + 3NaF → Na_3_Co_2_(PO_4_)_2_F_3_
Mn 1	Mn_2_O_3_ + 2(NH_4_)H_2_PO_4_ + 3NaF → Na_3_Mn_2_(PO_4_)_2_F_3_ + 3H_2_O + 2NH_3_
Mn 2	2(MnPO_4_·H_2_O) + 3NaF → Na_3_Mn_2_(PO_4_)_2_F_3_ + 2H_2_O
Ni/O_2_	Ni_2_P_2_O_7_ + 3NaF + 1/2O_2_ → Na_3_Ni_2_(PO_4_)_2_F_3_
Nb	2NbOPO_4_ + 3NaF → Na_3_Nb_2_(PO_4_)_2_F_3_ + O_2_
Nb/H_2_	2NbOPO_4_ + 3NaF + 2H_2_ → Na_3_Nb_2_(PO_4_)_2_F_3_ + 2H_2_O
Mo	2MoOPO_4_ + 3NaF → Na_3_Mo_2_(PO_4_)_2_F_3_ + O_2_
Mo/H_2_	2MoOPO_4_ + 3NaF + 2H_2_ → Na_3_Mo_2_(PO_4_)_2_F_3_ + 2H_2_O

## 4 Conclusion

An in-depth investigation was performed for the Na_*x*_VPF structure with vanadium and potential substituents from the first and second rows of transition metals. r^2^SCAN with D4 dispersion and Hubbard corrections was used to expand upon existing GGA + U-level studies. We observe improved agreement with experimental voltage plateaus for Na_*x*_VPF, decreasing the underestimation from 0.36–0.50 V for GGA + U to 0.28–0.04 for r^2^SCAN + U. Vanadium was substituted with Ti, Mn, Cr, Fe, Ni, Co, Mo, Zr and Nb. The second row transition metals exhibit a voltage that is about 1.5–2.4 V below their first row transition metal counterparts. Our model shows results that are comparable to the experimental ones as can be verified for Cr, Ti and Fe. However, a slight underestimation of the experimental voltage values remain, which can be related to imperfect Hubbard U corrections. Most substituents lead to too high voltages for practical operation, however Mn and Mo show very promising voltage plateaus for a Na exchange of up to 3 Na per f.u. The variation of the volume for the Na_*x*_VPF can be reproduced as expected from experiments. In the Na_*x*_MPF substituted materials with M = Ti, Fe, Cr, Mo, and Nb, the volume change is even reduced. However, the structures substituted with Mn and Ni exhibit non-negligible volume contraction, suggesting potentially an inferior cycling stability. The orthorhombicity analysis shows a consistently low *b/a* distortion for Ti, Fe, Mo and Cr across almost all Na concentrations, comparable to the NVPF cathode, whereas Nb exhibits larger distortion. Mn and Ni do exhibit a significant distortion on top of their volume contraction. The NEB calculations demonstrate that migration barriers are strongly influenced by both the transition metals and Na content, with lower barriers observed consistently for Mo-substituted structures. In contrast, Fe and Mn display a mixed influence depending on the state of charge. We finally proposed synthesis routes for our substituted materials; the Gibbs free energies of reaction for the synthesis of Na_3_MPF with M = Co, Cr, Mo, Mn, and Fe can be negative, indicating that these compounds can potentially be synthesized. In particular the Mn- and Mo-based materials appear particularly promising in terms of voltage curve, and with the proposed synthesis routes they may be experimentally realized. Considering abundance and cost, Mn appears to be a more commercially viable option, whereas Mo remains of great interest from a performance perspective, but abundance and cost need to be carefully assessed.

## Conflicts of interest

There are no conflicts to declare.

## Supplementary Material

TA-013-D5TA04213E-s001

## Data Availability

Data for this article, including formation energy for NVPF, voltage values, reaction energies, cell parameters, and NEB barriers are available at Zenodo at [URL-DOI https://doi.org/10.5281/zenodo.15507496] Supplementary information is available: includes the benchmarked U values for Nb and Mo; Na configurations for Na_1_V_2_(PO_4_)_2_F_3_, Na_2_V_2_(PO_4_)_2_F_3_, and Na_3_V_2_(PO_4_)_2_F_3_; voltage profile, cell parameters, and Gibbs free energy for Zr; DOS of V-, Mn-, Cr-, Co-, Fe-, and Mo-based materials; M−O and M−F bond distances for Na_x_M_2_(PO_4_)_2_F_3_ (0 ≤ *x* ≤ 4) where M = V, Ti, Cr, Mn, Fe, Co, Ni, Mo, and Nb; top-down view of Na-ion migration pathways in Na_0_V_2_(PO_4_)_2_F_3_; and Gibbs free energy for different synthesis routes including phonon calculations for Mn, Ti, and Fe. See DOI: https://doi.org/10.1039/d5ta04213e.
