# Supplementary material for: Computational design of phosphate fluoride cathode materials for Na-based batteries
Source: J Mater Chem A Mater. 2025 Sep 15;13(41):35521–32. doi: 10.1039/d5ta04213e (PMC12452074; doi:10.1039/d5ta04213e)
Supplement: TA-013-D5TA04213E-s001 [file TA-013-D5TA04213E-s001.pdf]

Supplementary Information (SI) for Journal of Materials Chemistry A.
This journal is © The Royal Society of Chemistry 2025

Supplementary Information

Table S1: ISCD references for all compounds.

Compound	ICSD Reference Number
Mn_2O_3	9091
$MnPO_4{\cdot}H_2O$	29536
$NbOPO_4$	24110
$FePO_4$	14971
$MoOPO_4$	24894
$(ZrO)_2P_2O_7$	1922
$Ni_2P_2O_7$	100194
$TiPO_4$	36520
$CrPO_4$	60836
$CoPO_4$	246224
NaF	29128
Na_1VPF	238514
$(NH_4)H_2PO_4$	192625
VPO_4	36521
Na_3VPF	149512
MoO_3	35076
MoO_2	23722
Mo	52267
Nb	76011
NbO	14338
Nb_2O_5	71317
NbO_2	645141
Na	159431

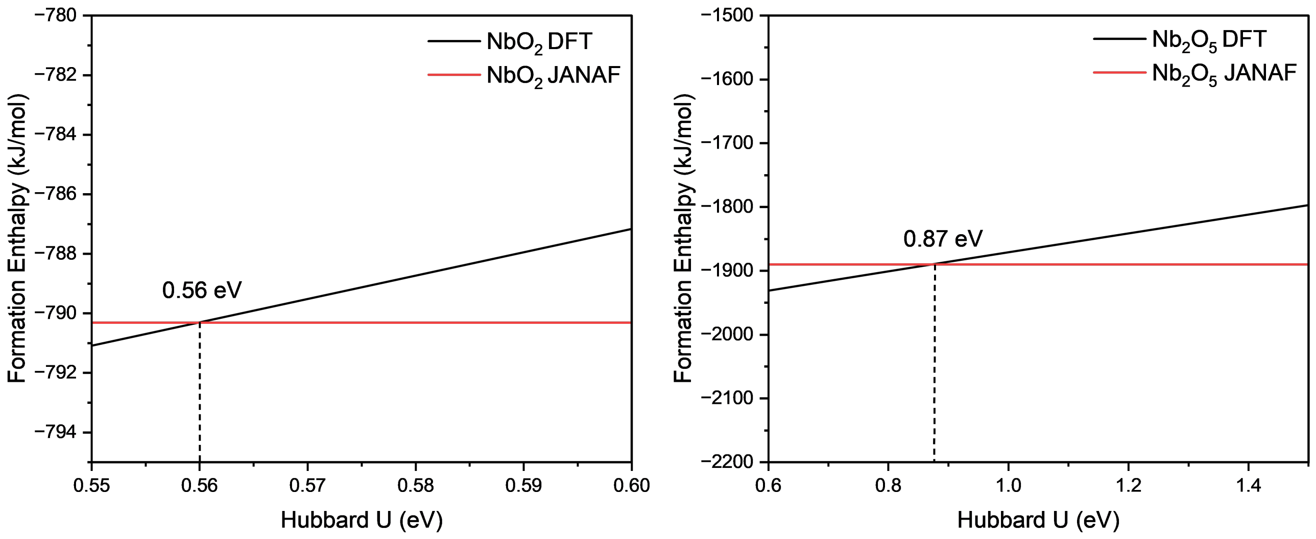

Figure S1: Benchmarking the Hubbard U value for Nb using NbO_2 and Nb_2O_5 within the DFT+U calculations: Comparison of calculated formation enthalpies with JANAF thermochemical data based on the reactions: Nb(s) + O_2(g)→ NbO_2; 2 Nb(s) + $\frac{5}{2}$ O_2(g) → Nb_2O_5, the U value of 0.71 used in this work represents the average of the individually benchmarked U value of NbO_2 and Nb_2O_5

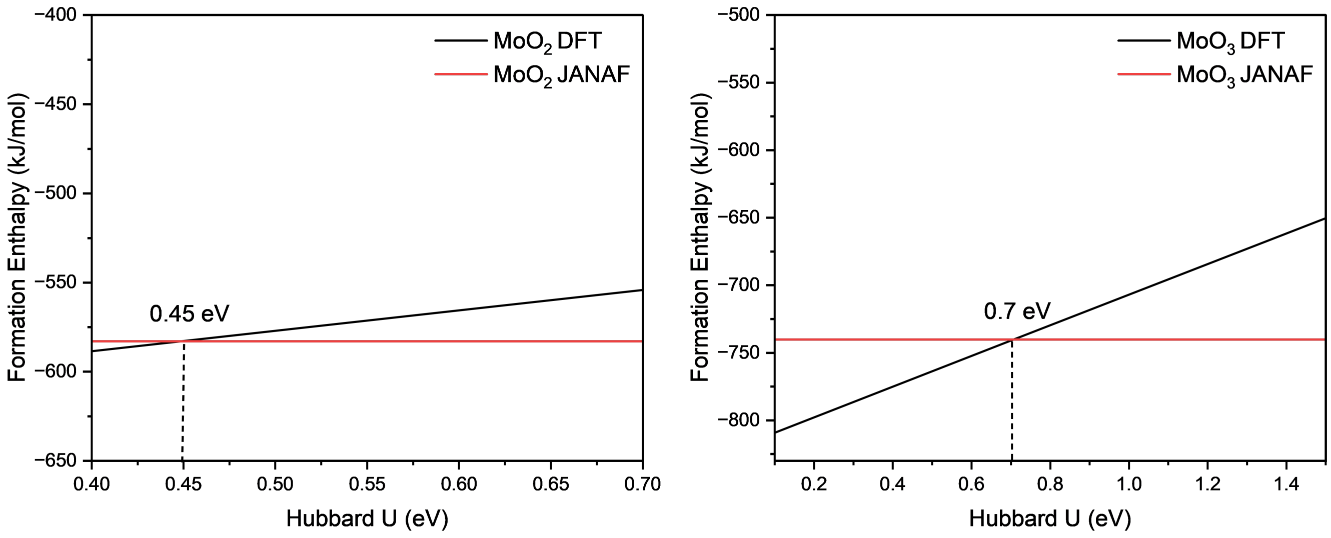

Figure S2: Benchmarking the Hubbard U value for Mo within MoO_2 and MoO_3 using the DFT+U calculations: Comparison of calculated formation enthalpies with JANAF thermochemical table based on the reactions: Mo(s) + O_2(g)$\rightarrow$ MoO_2; Mo(s) + $\frac{3}{2}$ O_2(g)$\rightarrow$ MoO_3, the U value of 0.57 used in this work represents the average of the individually benchmarked U value of MoO_2 and MoO_3

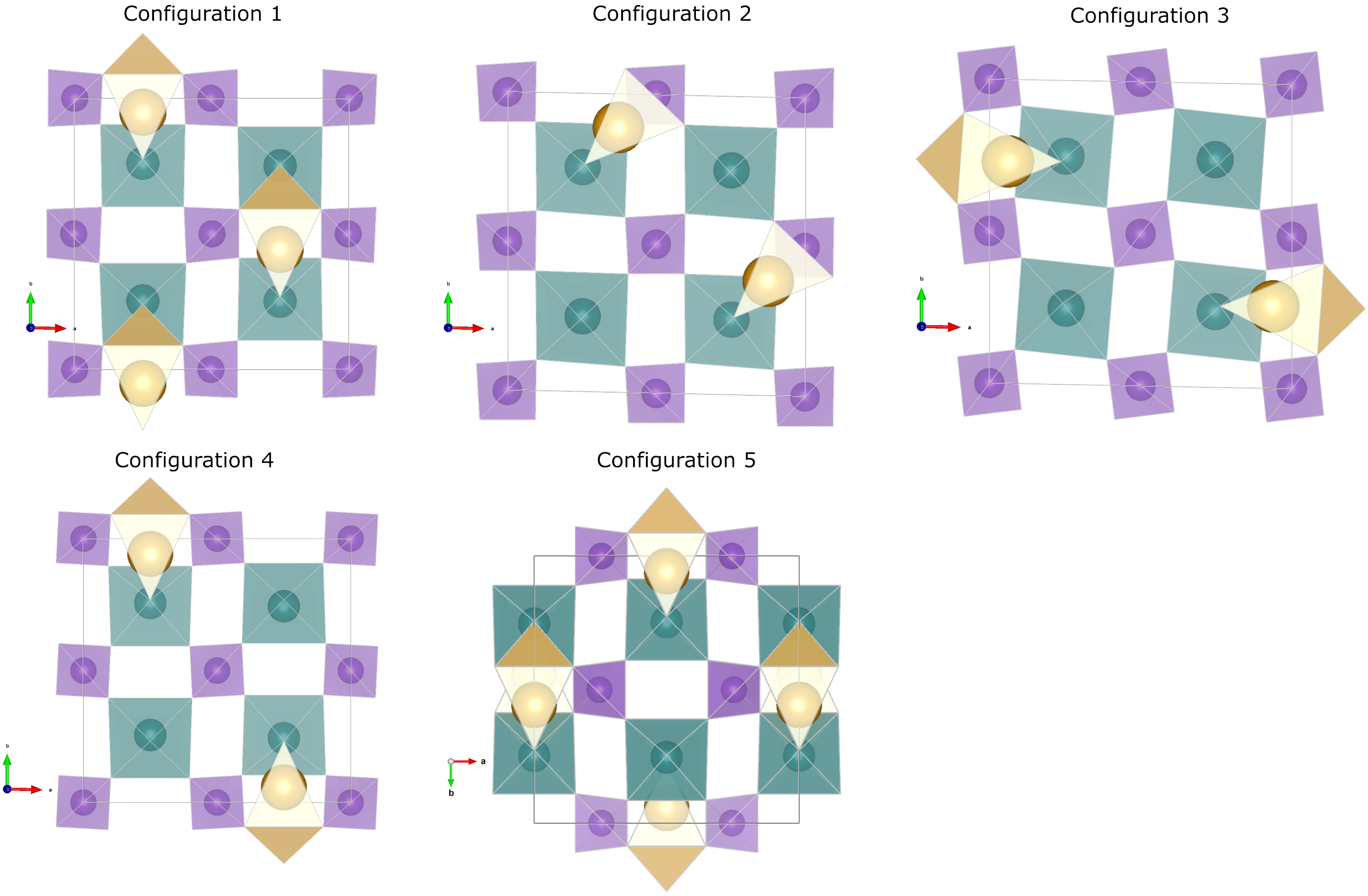

Figure S3: Illustration of the four Na configurations considered for $Na_1V_2(PO_4)_2F_3$, investigated using density functional theory (DFT), emphasizing distinct sodium arrangements. The configurations from 1 to 4 were created manually, while configuration 5 corresponds to experimental structure reported by Bianchini et al., as explained in the manuscript. Formation energies were calculated to investigate their stability, and the resulting values were used to construct the convex hull. The most stable configuration identified through this analysis was subsequently used for voltage calculation.

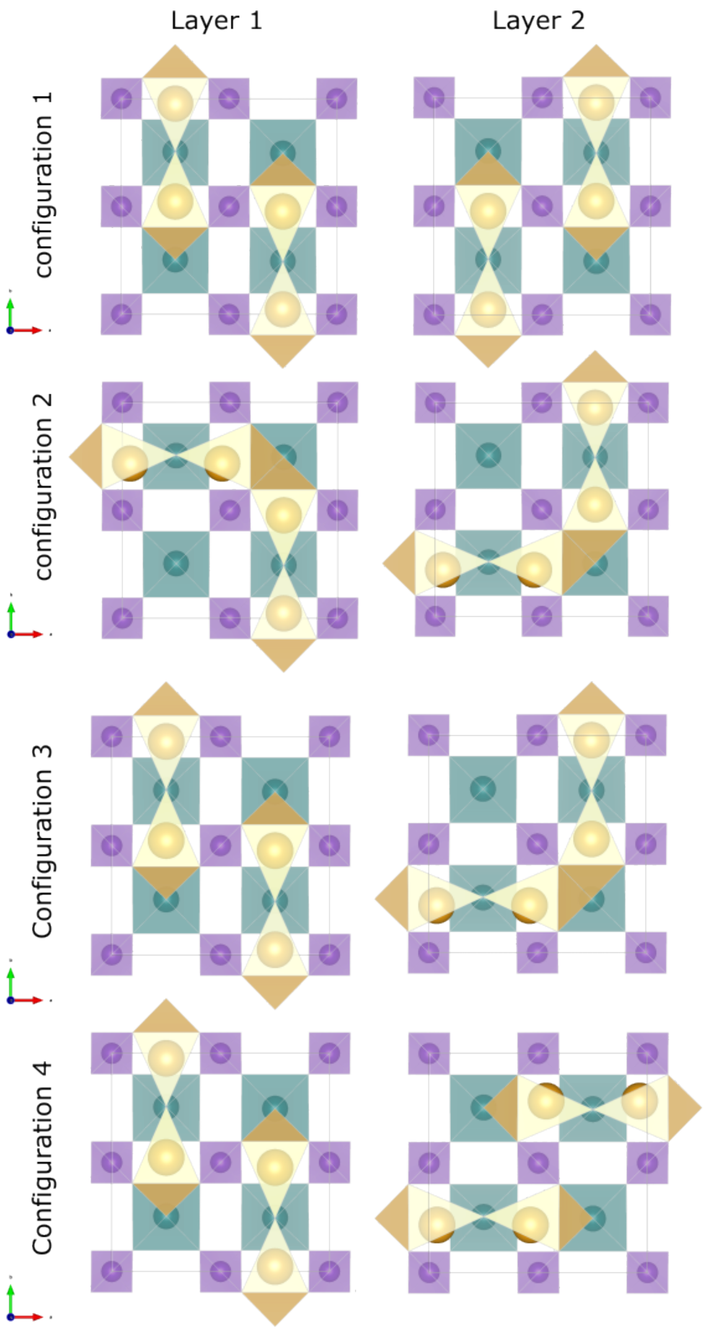

Figure S4: Illustration of the four Na configurations considered for $Na_2V_2(PO_4)_2F_3$, investigated using density functional theory (DFT). The figure shows layers 1 and 2 of each configuration, emphasizing distinct sodium arrangements. Formation energies were calculated to investigate their stability, and the resulting values were used to construct the convex hull. The most stable configuration identified through this analysis was subsequently used for voltage calculation.

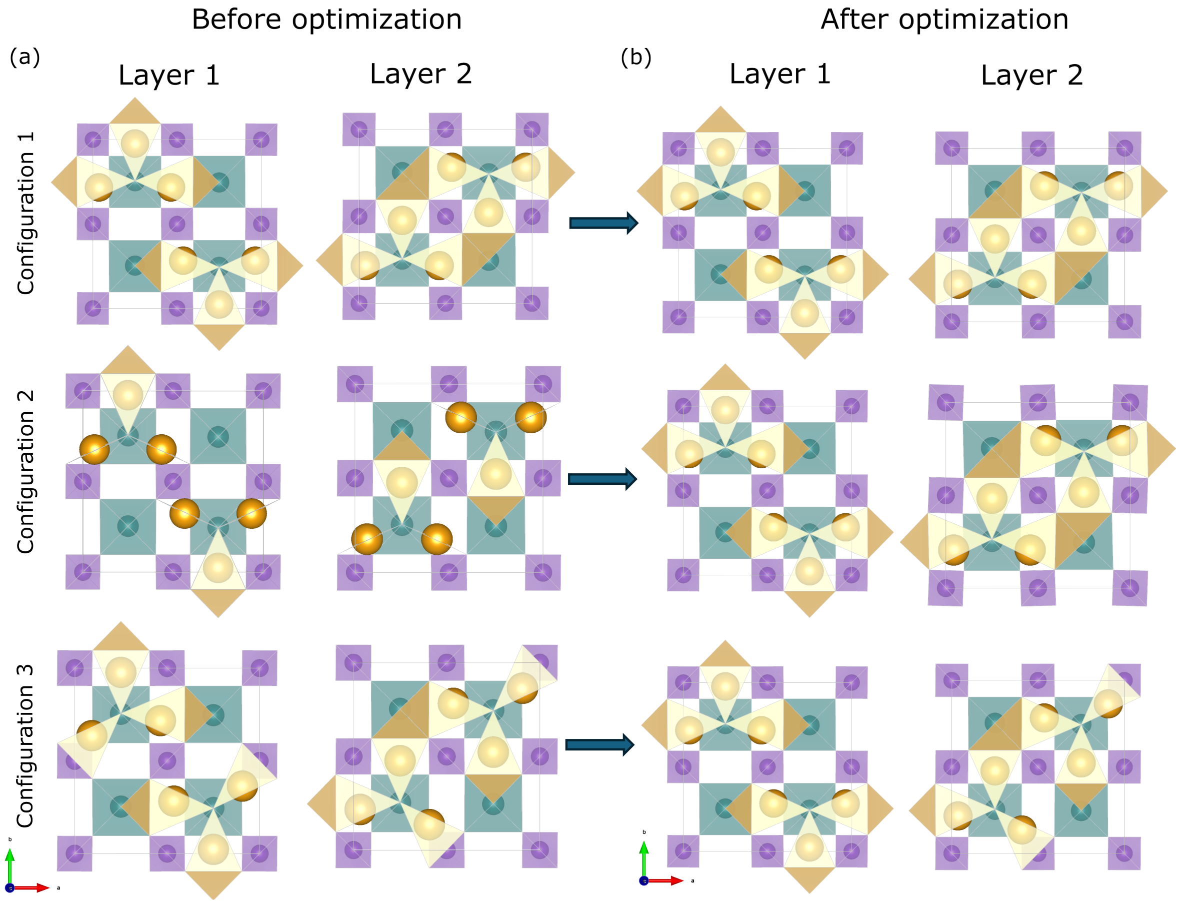

Figure S5: Illustration of layers 1 and 2 of the three configurations considered for $Na_3V_2(PO_4)_2F_3$,as illustrated in the figure (a): before optimization and (b): after the optimization, each showing distinct Na-ion arrangements within the structure. These configurations were investigated using density functional theory (DFT), and their formation energies were calculated to assess their stability. The resulting energies were used in the construction of the convex hull to identify the most stable configuration, which was then used for voltage calculations.

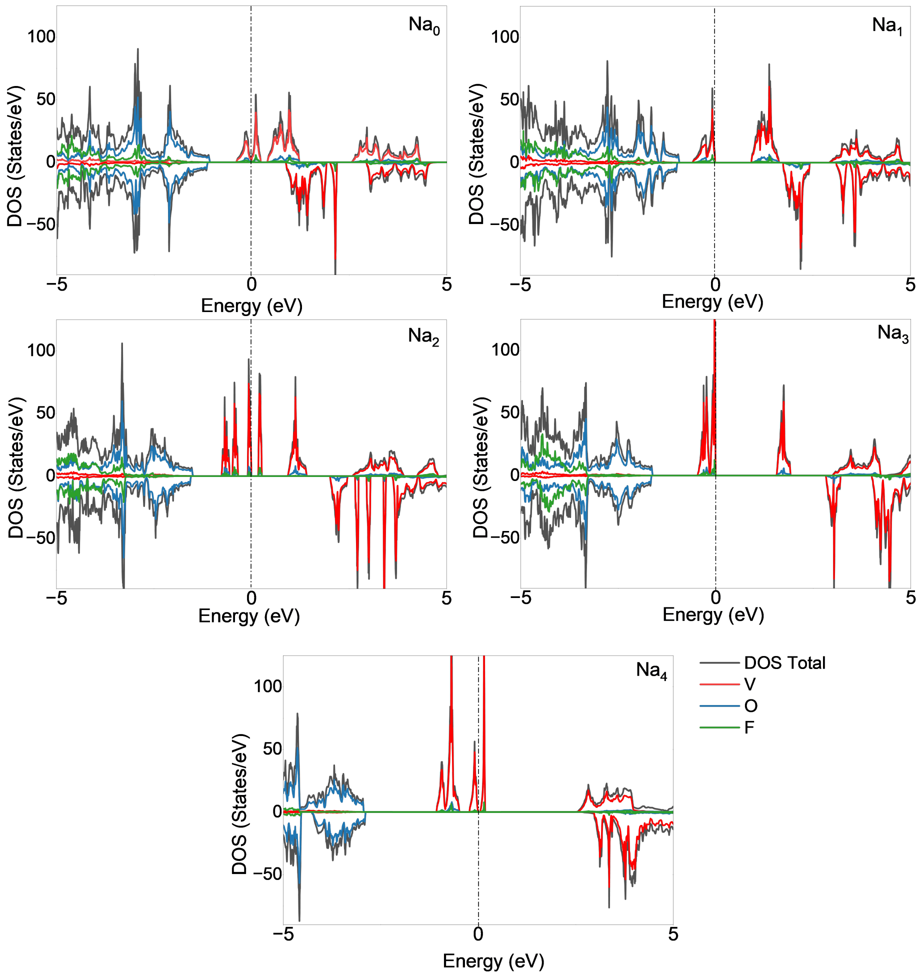

Figure S6: Total and projected density of states (DOS and pDOS) for V (red), F (green) and O (blue) for $Na_xV_2(PO_4)_2F_3$ compound, shown for Na concentrations from Na_0 to Na_4 (from top to bottom).

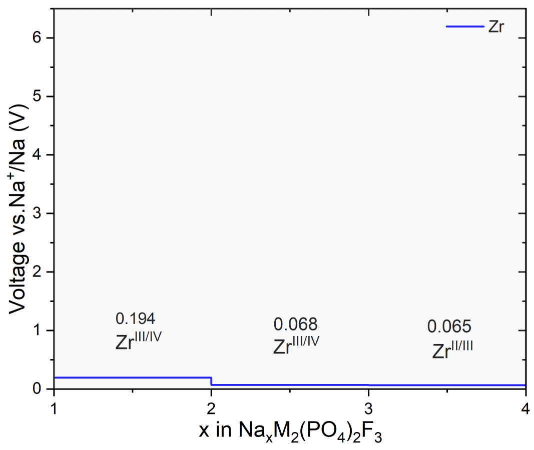

Figure S7: Voltage profile of $Na_xZr_2(PO_4)_2F_3$ using r^{2}s2SCAN functional without applying Hubbard correction.

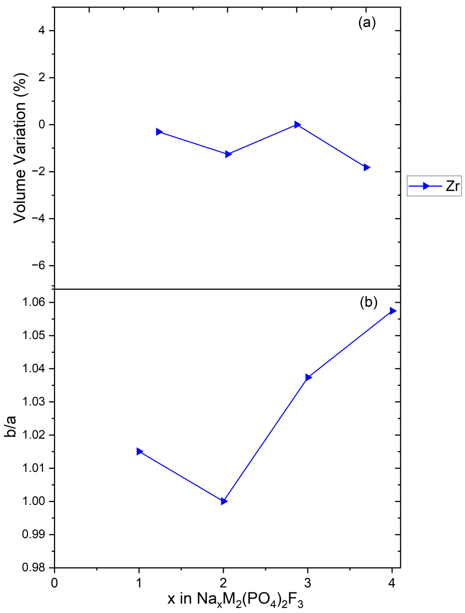

Figure S8: Panel (a): Unit cell volume and panel (b): Unit cell parameters ratio a/b for $Na_xZr_2(PO_4)_2F_3$ based on DFT calculation, using r^2s2SCAN functional without applying Hubbard correction.

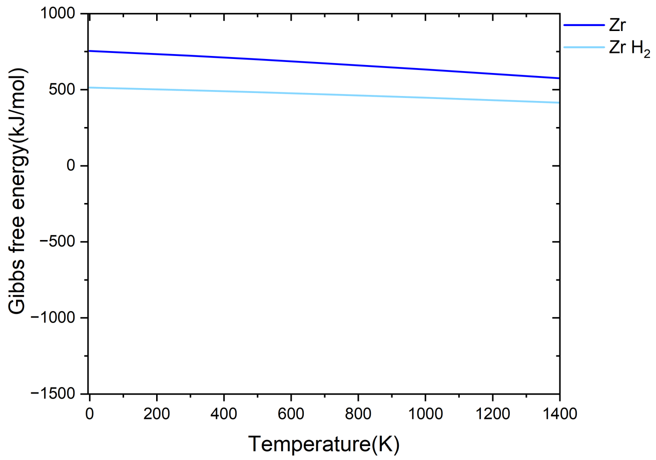

Figure S9: Gibbs free energy as a function of temperature for $Na_3Zr_2(PO_4)_2F_3$, based on two synthesis routes. The first route (dark blue): $(ZrO)_2P_2O_7 + 3NaF \rightarrow Na_3Zr_2(PO_4)_2F_3 + \frac{1}{2}O_2$, and the second route using a reducing agent the H_2 (light blue): $(ZrO)_2P_2O_7 + 3NaF + H_2 \rightarrow Na_3Zr_2(PO_4)_2F_3 + H_2O$.

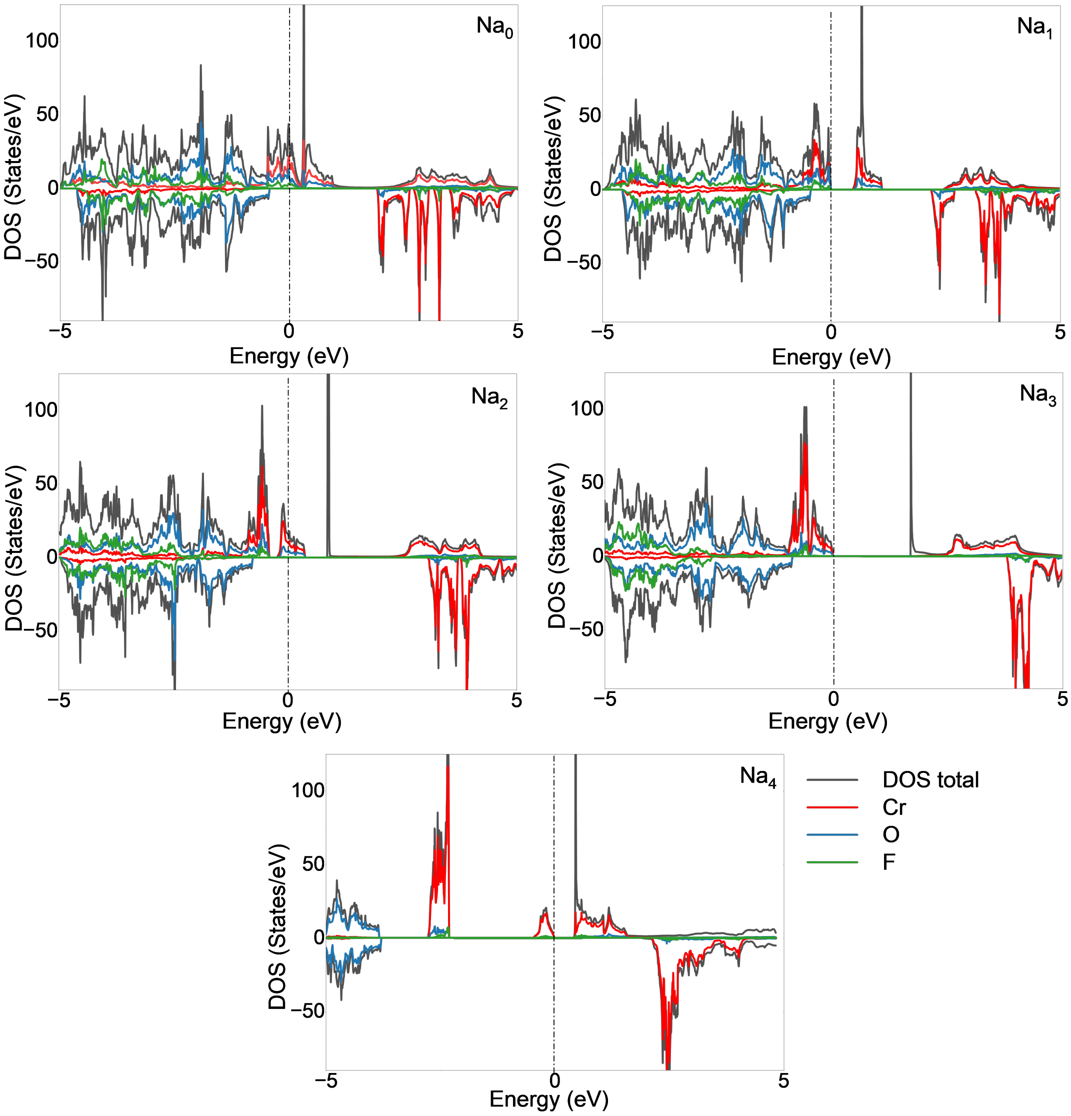

Figure S10: Total and projected density of states(DOS and pDOS) for Cr (red), F (green) and O (blue) for $Na_xCr_2(PO_4)_2F_3$ compound, shown for Na concentrations from Na_0 to Na_4 (from top to bottom).

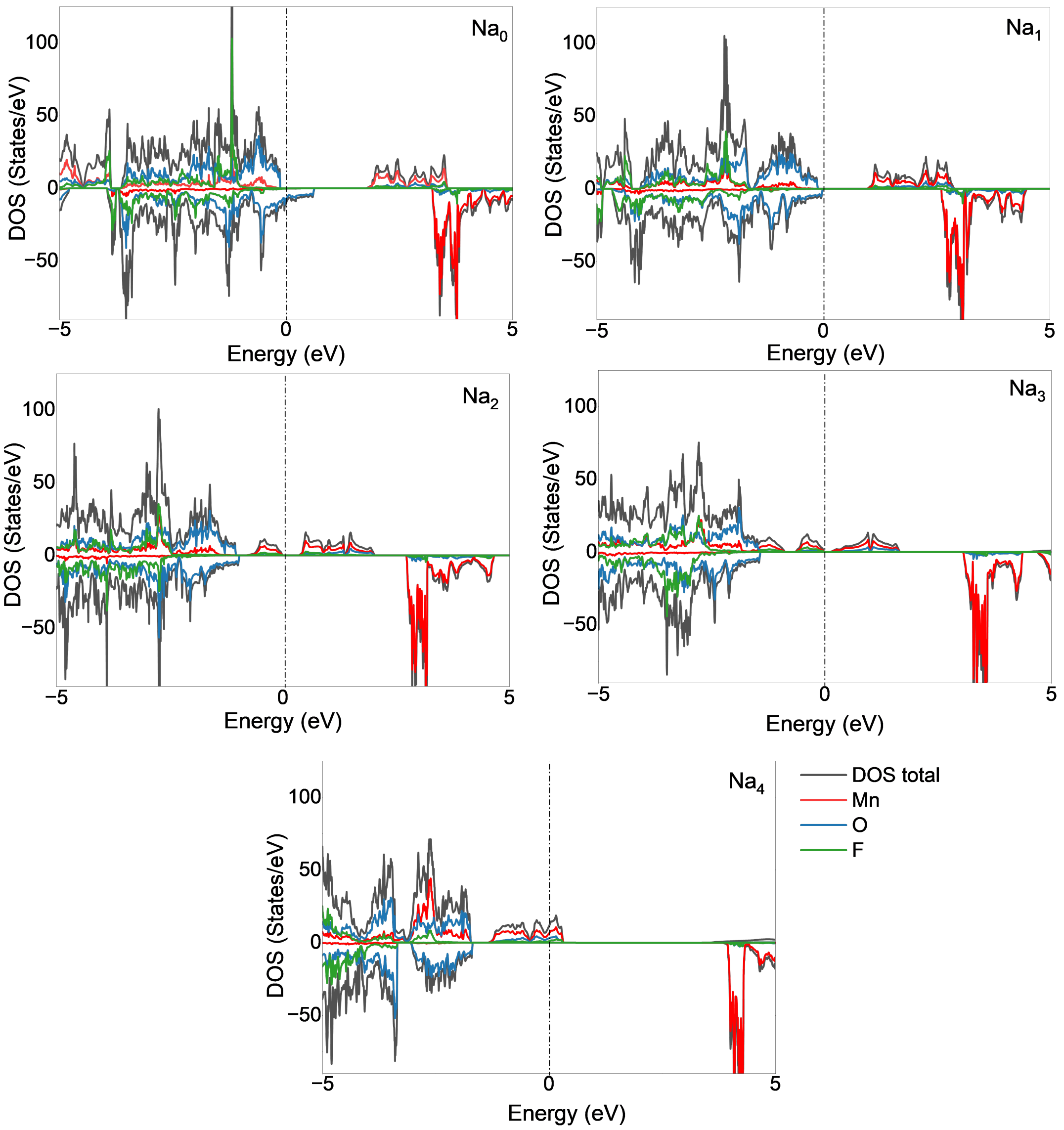

Figure S11: Total and projected density of states(DOS and pDOS) for Mn (red), F (green) and O (blue) for $Na_xMn_2(PO_4)_2F_3$ compound, shown for Na concentrations from Na_0 to Na_4 (from top to bottom).

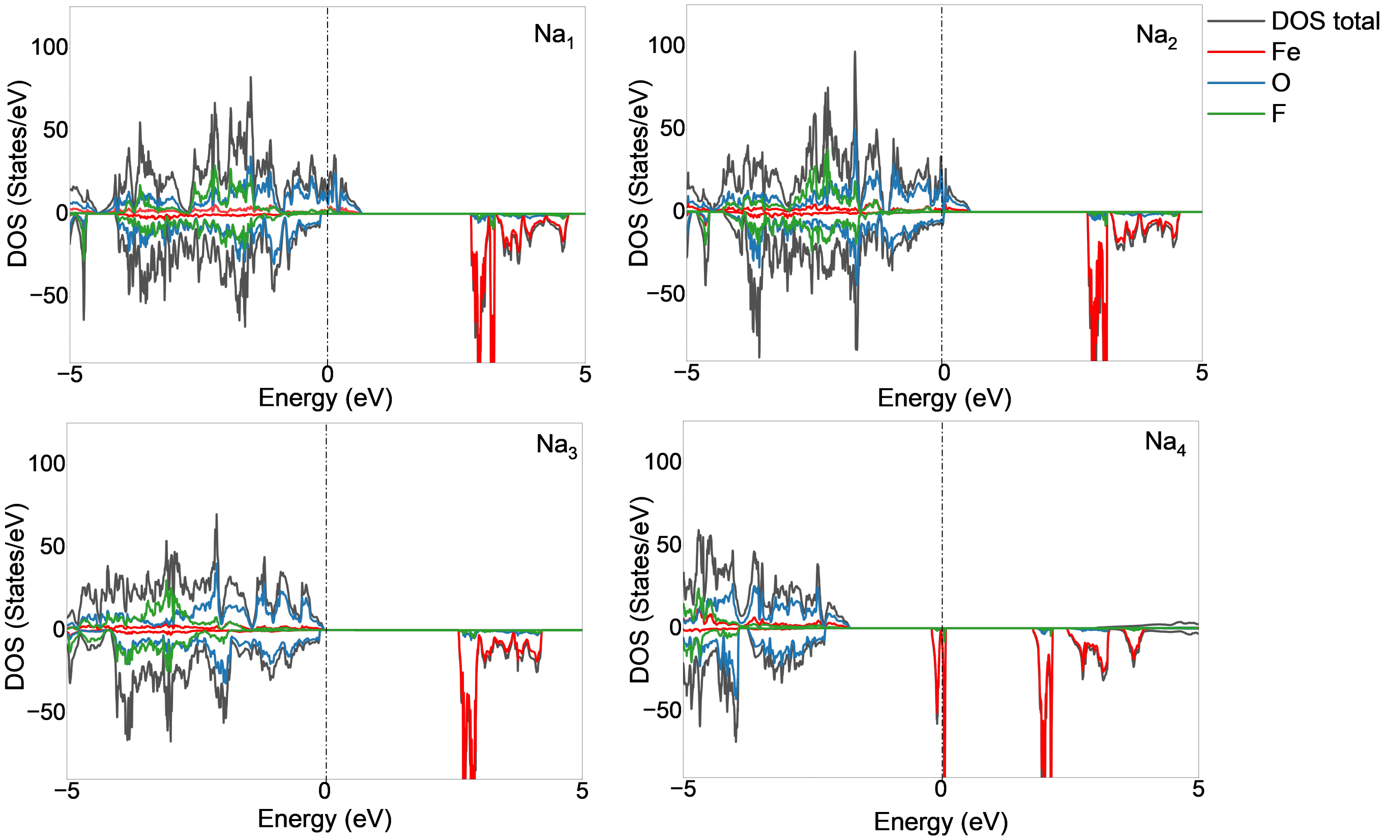

Figure S12: Total and projected density of states(DOS and pDOS) for Fe (red), F (green) and O (blue) for $Na_xFe_2(PO_4)_2F_3$ compound, shown for Na concentrations from Na_1 to Na_4 (from top to bottom).

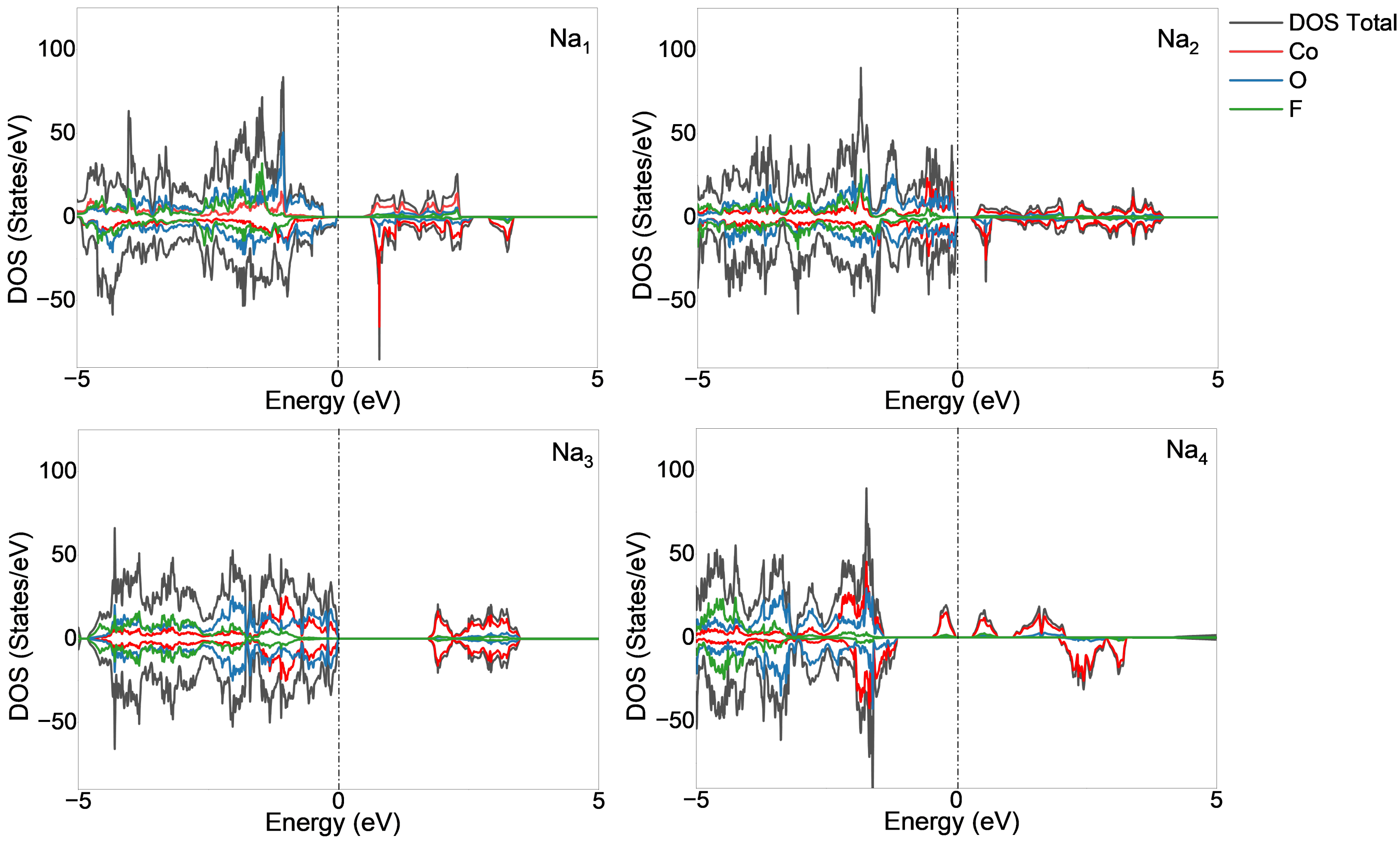

Figure S13: Total and projected density of states(DOS and pDOS) for Co (red), F (green) and O (blue) for $Na_xCo_2(PO_4)_2F_3$ compound, shown for Na concentrations from Na_1 to Na_4 (from top to bottom).

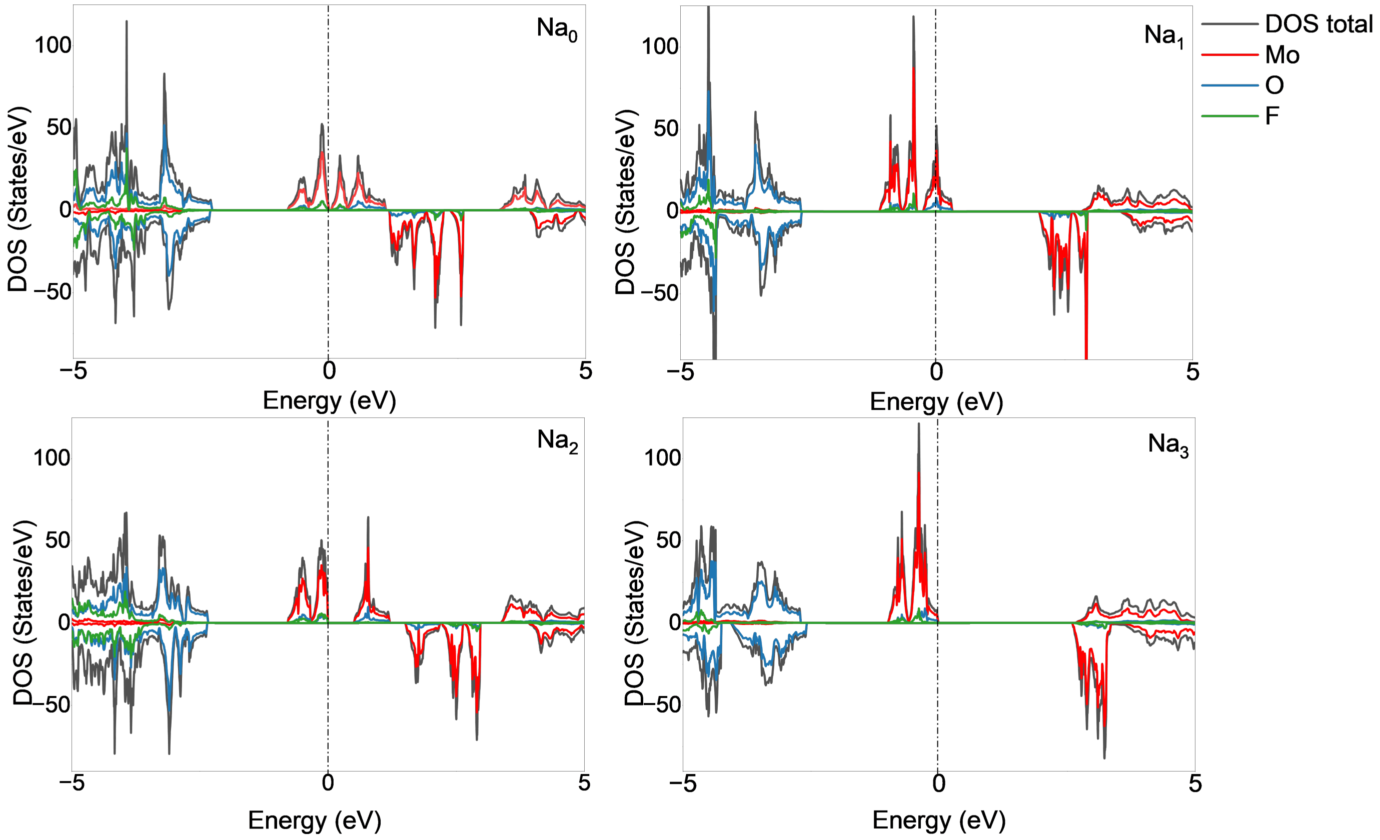

Figure S14: Total and projected density of states(DOS and pDOS) for Mo (red), F (green) and O (blue) for $Na_xMo_2(PO_4)_2F_3$compound, shown for Na concentrations from Na_0 to Na_3 (from top to bottom).

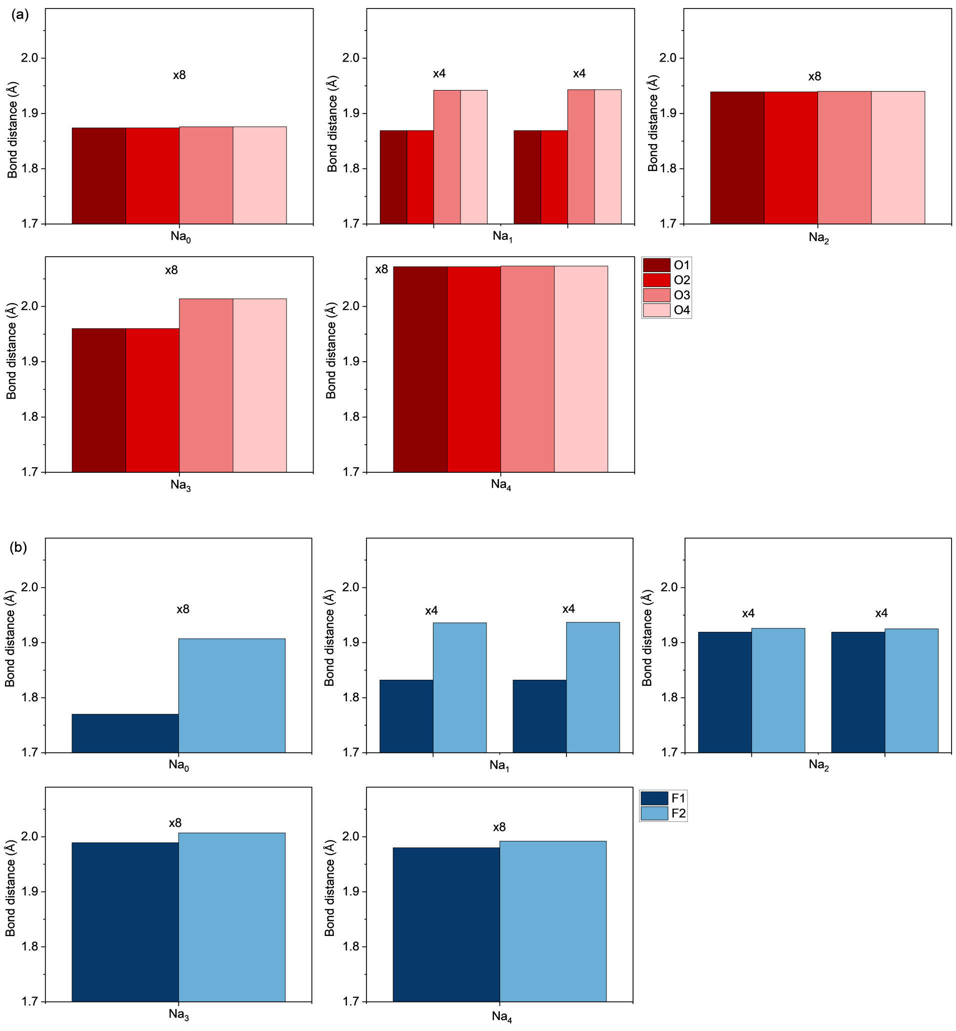

Figure S15: M-O and M-F bond distances in the eight octahedra of $Na_xV_2(PO_4)_2F_3$ with Na content from x = 0 to 4. Panel (a) shows M-O distances involving four oxygen atoms (O1, O2, O3, O4), while panel (b) displays axial M-F distances involving two fluorine atoms (F1, F2). When multiple octahedra share identical distances, multiplicities are indicated explicitly (e.g.,” $\times$ 4” for four identical octahedra or ” $\times$ 8” when all are identical).

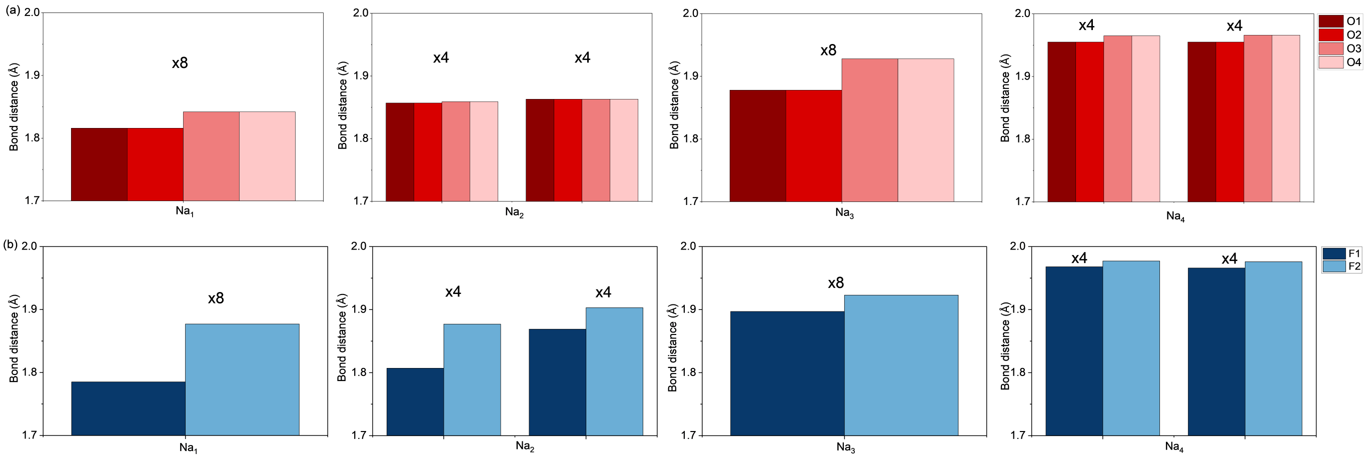

Figure S16: M-O and M-F bond distances in the eight octahedra of $Na_xCo_2(PO_4)_2F_3$ with Na content from x = 1 to 4. Panel (a) shows M-O distances involving four oxygen atoms (O1, O2, O3, O4), while panel (b) displays axial M-F distances involving two fluorine atoms (F1, F2). When multiple octahedra share identical distances, multiplicities are indicated explicitly (e.g.,” $\times$ 4” for four identical octahedra or ” $\times$ 8” when all are identical).

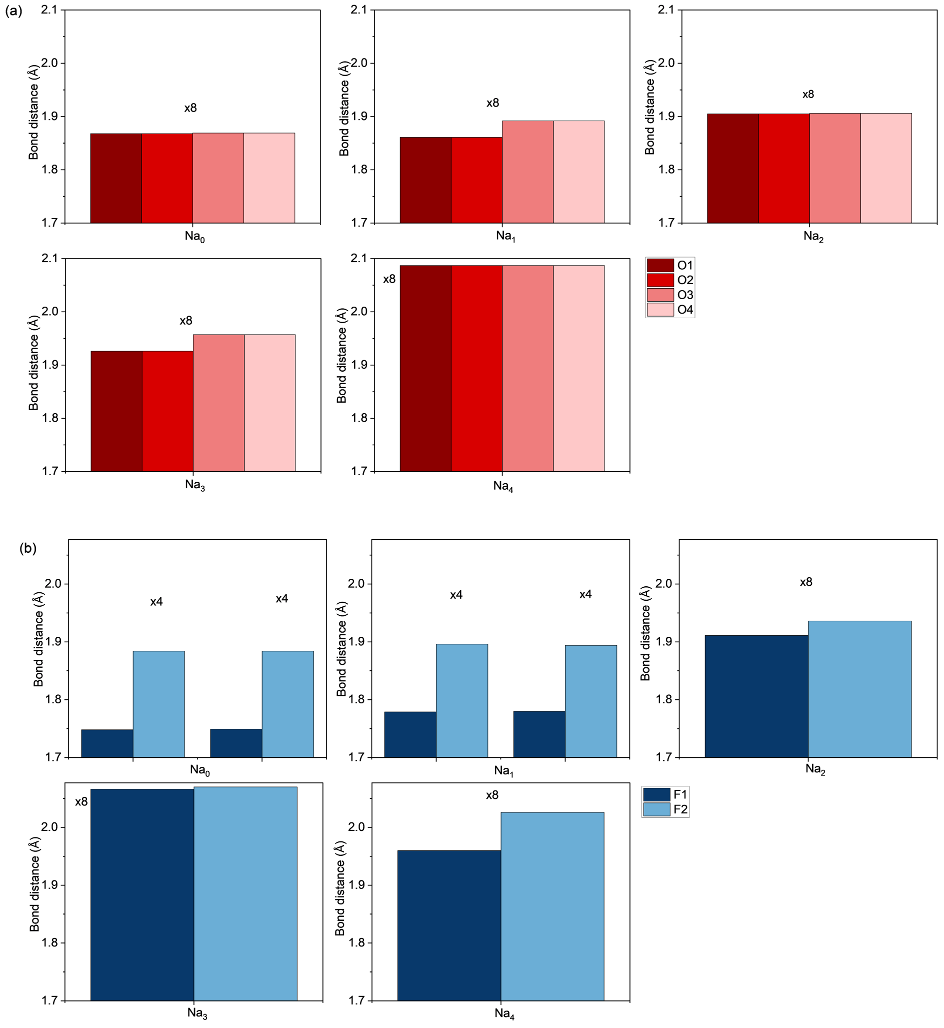

Figure S17: M-O and M-F bond distances in the eight octahedra of $Na_xMn_2(PO_4)_2F_3$ with Na content from x = 0 to 4. Panel (a) shows M-O distances involving four oxygen atoms (O1, O2, O3, O4), while panel (b) displays axial M-F distances involving two fluorine atoms (F1, F2). When multiple octahedra share identical distances, multiplicities are indicated explicitly (e.g.," $\times$ 4" for four identical octahedra or " $\times$ 8" when all are identical).

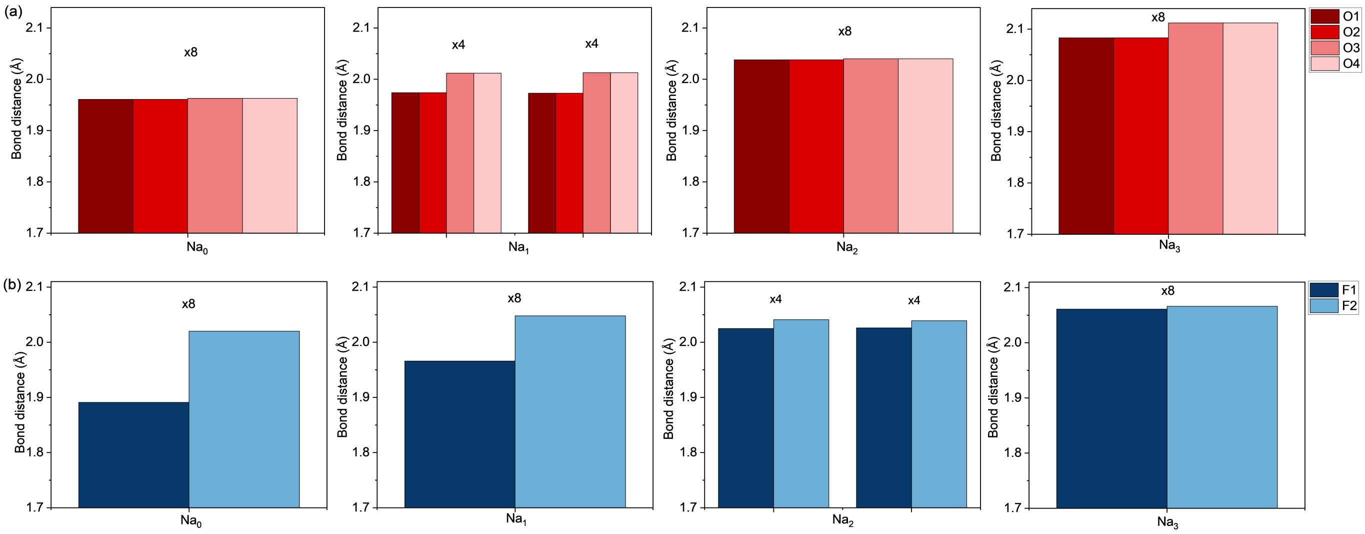

Figure S18: M-O and M-F bond distances in the eight octahedra of $Na_xMo_2(PO_4)_2F_3$ with Na content from x = 0 to 3. Panel (a) shows M-O distances involving four oxygen atoms (O1, O2, O3, O4), while panel (b) displays axial M-F distances involving two fluorine atoms (F1, F2). When multiple octahedra share identical distances, multiplicities are indicated explicitly (e.g.," $\times$ 4" for four identical octahedra or " $\times$ 8" when all are identical).

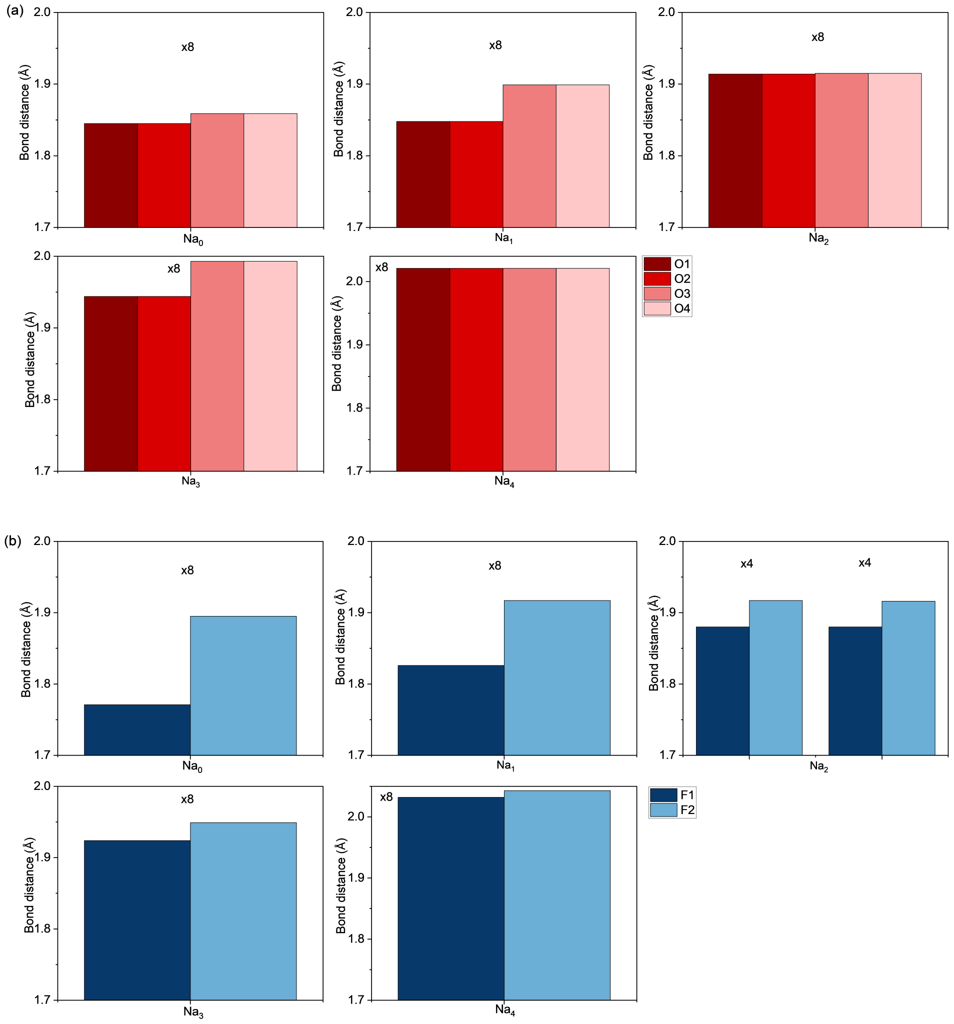

Figure S19: M-O and M-F bond distances in the eight octahedra of$Na_xCr_2(PO_4)_2F_3$ with Na content from x = 0 to 4. Panel (a) shows M-O distances involving four oxygen atoms (O1, O2, O3, O4), while panel (b) displays axial M-F distances involving two fluorine atoms (F1, F2). When multiple octahedra share identical distances, multiplicities are indicated explicitly (e.g.," × 4" for four identical octahedra or" × 8" when all are identical).

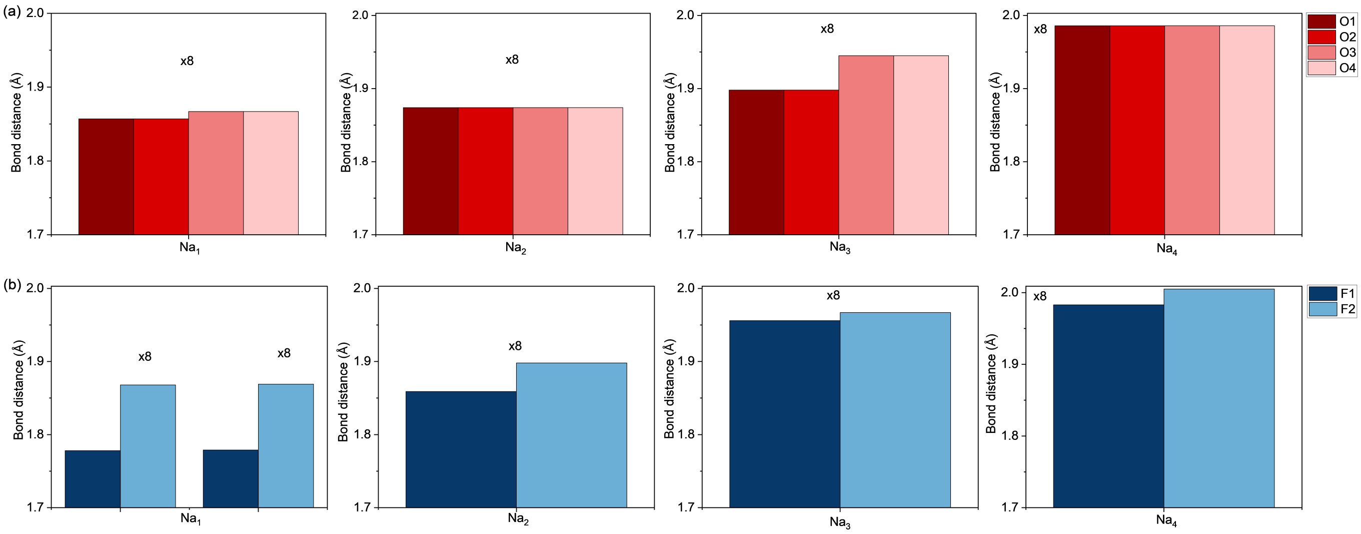

Figure S20: M-O and M-F bond distances in the eight octahedra of $Na_xNi_2(PO_4)_2F_3$ with Na content from x = 1 to 4. Panel (a) shows M-O distances involving four oxygen atoms (O1, O2, O3, O4), while panel (b) displays axial M-F distances involving two fluorine atoms (F1, F2). When multiple octahedra share identical distances, multiplicities are indicated explicitly (e.g.," $\times$ 4" for four identical octahedra or " $\times$ 8" when all are identical).

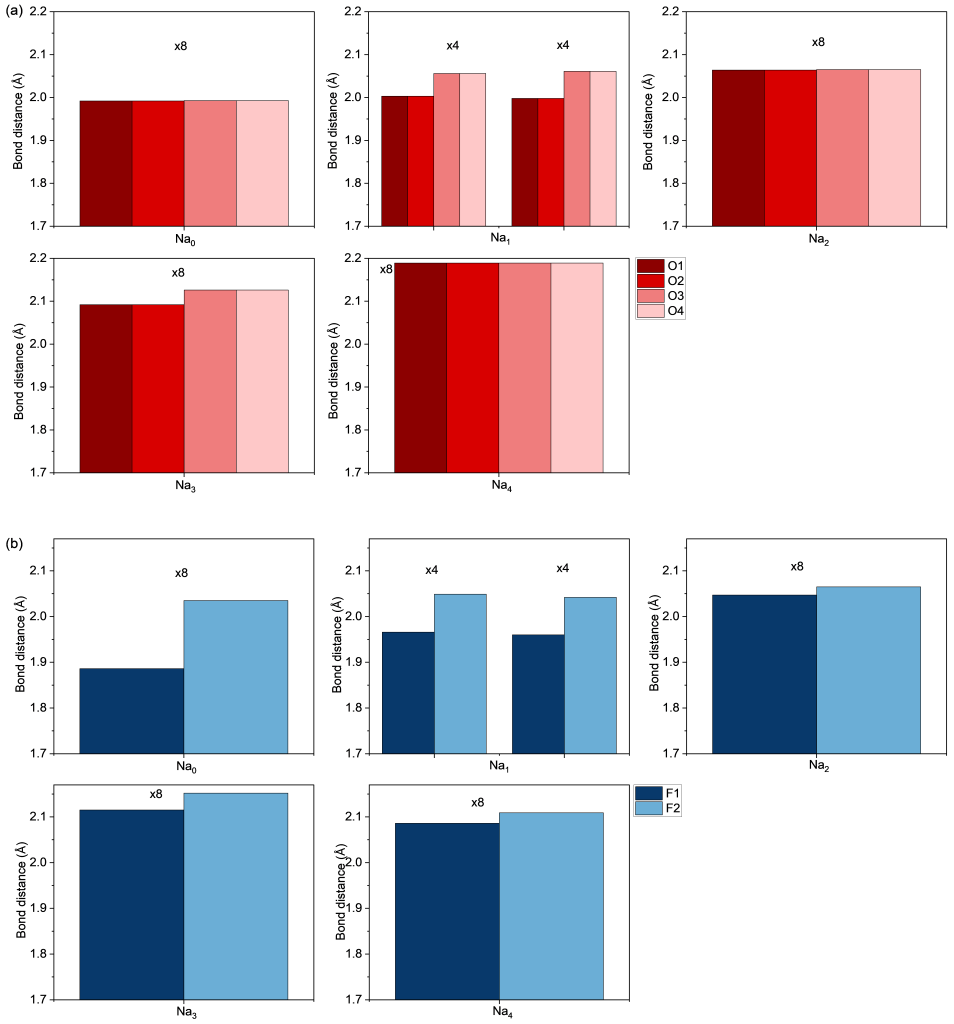

Figure S21: M-O and M-F bond distances in the eight octahedra of $Na_xNb_2(PO_4)_2F_3$ with Na content from x = 0 to 4. Panel (a) shows M-O distances involving four oxygen atoms (O1, O2, O3, O4), while panel (b) displays axial M-F distances involving two fluorine atoms (F1, F2). When multiple octahedra share identical distances, multiplicities are indicated explicitly (e.g.,” $\times$ 4” for four identical octahedra or ” $\times$ 8” when all are identical).

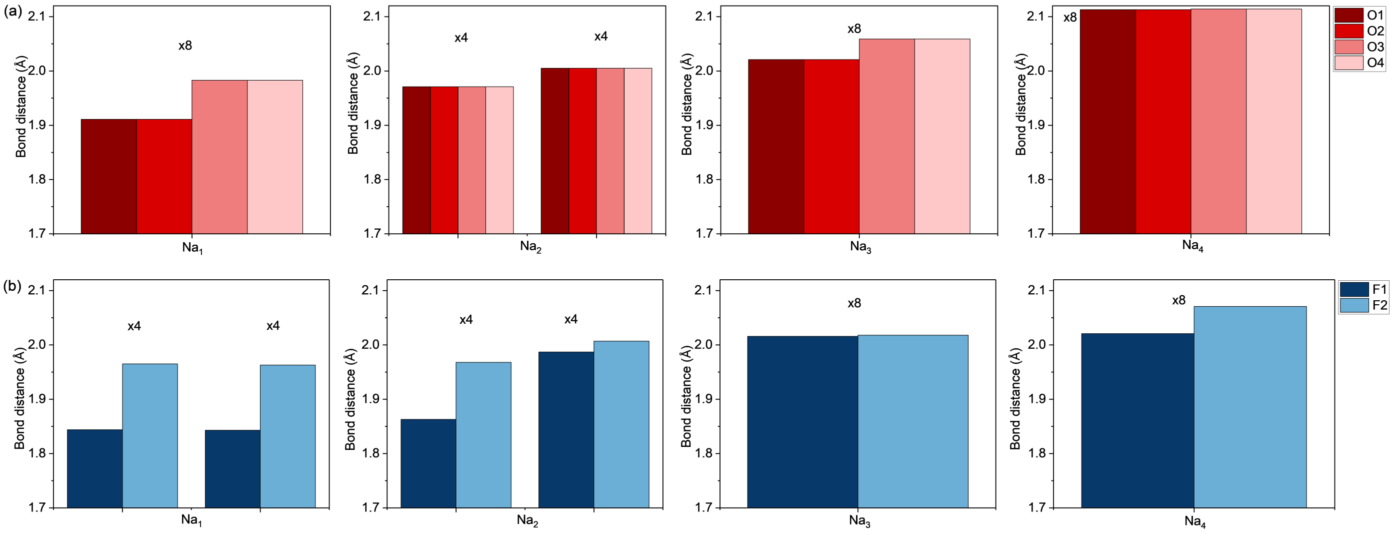

Figure S22: M-O and M-F bond distances in the eight octahedra of $Na_xTi_2(PO_4)_2F_3$ with Na content from x = 1 to 4. Panel (a) shows M-O distances involving four oxygen atoms (O1, O2, O3, O4), while panel (b) displays axial M-F distances involving two fluorine atoms (F1, F2). When multiple octahedra share identical distances, multiplicities are indicated explicitly (e.g.,” $\times$ 4” for four identical octahedra or ” $\times$ 8” when all are identical).

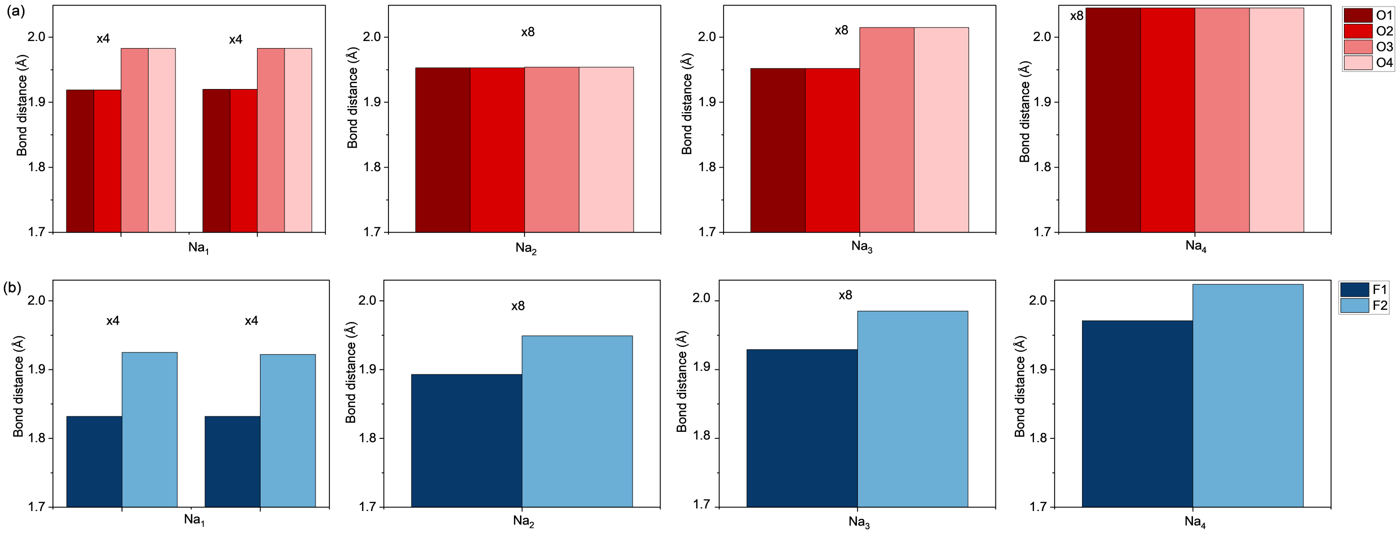

Figure S23: M-O and M-F bond distances in the eight octahedra of $Na_xFe_2(PO_4)_2F_3$ with Na content from x = 1 to 4. Panel (a) shows M-O distances involving four oxygen atoms (O1, O2, O3, O4), while panel (b) displays axial M-F distances involving two fluorine atoms (F1, F2). When multiple octahedra share identical distances, multiplicities are indicated explicitly (e.g.,” $\times$ 4” for four identical octahedra or ” $\times$ 8” when all are identical).

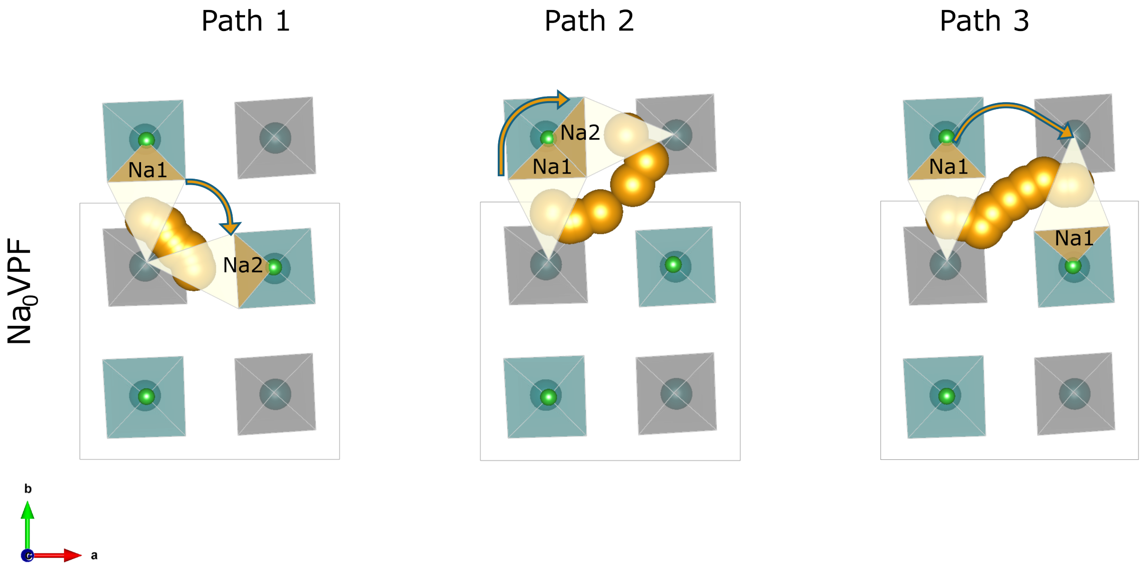

Figure S24: Top-down (*ab*-plane) view of Na-ion migration pathways in Na_0VPF, highlighting path 1 (intra-unit migration), path 2 (inter-unit migration between Na1 and Na2 sites), and path 3 (inter-unit migration between Na1 sites). The Na migration pathways are specified by yellow arrows. The green octahedra represent those with fluorine atoms (in bright green) in the same plane as the Na ions (at $z = 0$), while the grey ones correspond to those higher along the c axis (apical fluorine at $z = 0.127$, not shown).

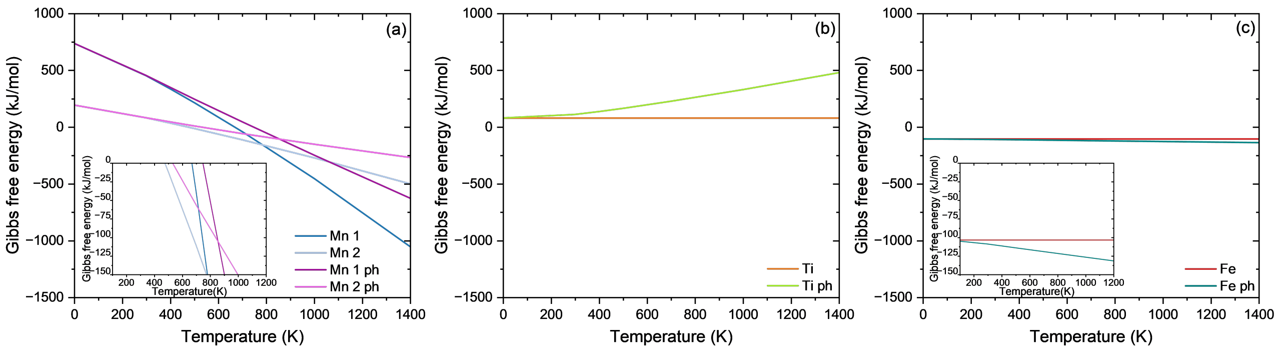

Figure S25: Gibbs free energy for different synthesis routes. (a): Mn 1 and Mn 2, with their phonon contributions (Mn 1 ph and Mn 2 ph), (b): Ti with and without phonon contributions (Ti and Ti ph),(c): Fe, with and without phonon contributions (Fe and Fe ph)
